# Methods of Weaning From Mechanical Ventilation in Adult: A Network Meta-Analysis

**DOI:** 10.3389/fmed.2021.752984

**Published:** 2021-10-04

**Authors:** Hong-Jie Jhou, Po-Huang Chen, Liang-Jun Ou-Yang, Chin Lin, Shih-En Tang, Cho-Hao Lee

**Affiliations:** ^1^Department of Neurology, Changhua Christian Hospital, Changhua, Taiwan; ^2^School of Medicine, Kaohsiung Medical University, Kaohsiung, Taiwan; ^3^Department of General Medicine, National Defense Medical Center, Tri-Service General Hospital, Taipei, Taiwan; ^4^Department of Internal Medicine, National Defense Medical Center, Tri-Service General Hospital, Taipei, Taiwan; ^5^Department of Physical Medicine and Rehabilitation, Chang Gung Memorial Hospital, Taoyuan, Taiwan; ^6^School of Public Health, National Defense Medical Center, Taipei, Taiwan; ^7^Department of Research and Development, National Defense Medical Center, Taipei, Taiwan; ^8^Division of Pulmonary and Critical Care Medicine, Department of Internal Medicine, National Defense Medical Center, Tri-Service General Hospital, Taipei, Taiwan; ^9^National Defense Medical Center, Graduate Institute of Aerospace and Undersea Medicine, Taipei, Taiwan; ^10^Division of Hematology and Oncology Medicine, Department of Internal Medicine, National Defense Medical Center, Tri-Service General Hospital, Taipei, Taiwan

**Keywords:** systemic review, network meta-analysis, weaning, T-piece, proportional assist ventilation, SmartCare

## Abstract

**Background/Objective:** The aim of study is to assess the efficacy of each ventilator weaning method for ventilated patients in intensive care units (ICUs).

**Methods:** A systematic search was conducted using PubMed, Embase, and China National Knowledge Infrastructure to identify randomized control studies on ventilated patients regarding extubation associated outcomes (weaning success or failure, proportion requiring re-intubation, or mortality) from inception until April 01, 2020. Commonly used ventilation modes involved pressure support ventilation, synchronized intermittent mandatory ventilation, automatic tube compensation, continuous positive airway pressure, adaptive support ventilation, neurally adjusted ventilatory assist, proportional assisted ventilation, and SmartCare. Pooled estimates regarding extubation associated outcomes were calculated using network meta-analysis.

**Results:** Thirty-nine randomized controlled trials including 5,953 patients met inclusion criteria. SmartCare and proportional assist ventilation were found to be effective methods in increasing weaning success (odds ratio, 2.72, 95% confidence interval (CI), 1.33–5.58, *P*-score: 0.84; odds ratio, 2.56, 95% CI, 1.60–4.11, *P*-score: 0.83; respectively). Besides, proportional assist ventilation had superior in reducing proportion requiring re-intubation rate (odds ratio, 0.48, 95% CI, 0.25–0.92, *P*-score: 0.89) and mortality (odds ratio, 0.48, 95% CI, 0.26–0.92, *P*-score: 0.91) than others.

**Conclusion:** In general consideration, our study provided evidence that weaning with proportional assist ventilation has a high probability of being the most effective ventilation mode for patients with mechanical ventilation regarding a higher rate of weaning success, a lower proportion requiring reintubation, and a lower mortality rate than other ventilation modes.

## Introduction

The most common cause of vital organ failure was acute respiratory failure in critically ill patients. It was estimated that 40–65% of patients in intensive care units (ICUs) needed mechanical ventilation (1), which provided adequate oxygen and reduced the work of breathing for patients with respiratory failure of different etiologies (2). However, there were several complications associated with mechanical ventilation, such as initiating lung injury, ventilator-associated pneumonia (3), and respiratory muscle weakness (4).

Successful and timely weaning of patients from mechanical ventilation could shorten the duration of the ventilation and reduce infection risk, medical costs, and mortality. Some evidence showed that delay weaning might cause unnecessary discomfort, increase complication rates, and result to higher medical costs (5, 6). Even in scheduled extubated patients, up to one-third of patients needed reintubation because of extubation failure (7, 8). Reintubation was associated with high mortality due to new complications (9).

A spontaneous breathing trial (SBT) was the most common method to evaluate the ability of a patient to self-breathing and provided important clinical information for weaning. According to the American Thoracic Society Clinical Practice Guidelines on weaning and extubation (10, 11), an initial SBT was weakly recommended for weaning. PSV and T-piece for SBT in adults were commonly used modes for the liberation process. In a Cochrane review (12), Ladeira et al. found non-significant differences between the pressure support and T-piece modes regarding weaning success, pneumonia, reintubation, ICU mortality, and length of hospital stay.

Closed-loop weaning systems, an automatic system using physiological feedback signal to adjust the process of weaning, may facilitate systematic and early identification of spontaneous breathing ability and the potential for ventilation discontinuation through continuous monitoring and real-time interventions. The concept of closed-loop weaning systems is not new; however, with advanced technology from academia and industry, SmartCare is the first commercial closed-loop systems with intelligent modes in clinical use, and adaptive support ventilation, neurally adjusted ventilatory assist, and proportional assisted ventilation (PAV) have been further developed in recent decades (13). In current studies, closed-loop weaning systems show clinical benefit regarding a reduction of duration of weaning, mechanical ventilation, and length of ICU stay (13, 14).

PAV was first introduced by Younes in 1992 and adjusted the inspiratory pressure in proportion to the flow and volume generated by the patient. New software (PAV+) has been developed based on PAV to adapt to clinical needs through semi-continuous measurements and delivering pressure proportional to the instantaneous inspiratory flow and volume (13, 14). In a meta-analysis (15), PAV+ had benefits of decreasing the rate of weaning failure and the duration of mechanical ventilation in comparison with pressure support ventilation. Another meta-analysis provided the evidence that PAV increases the rate of weaning success, decreases proportion of patients requiring reintubation and the length of ICU stay, but does not reduce the mortality in comparison with pressure support ventilation (16).

Several meta-analyses have evaluated different ventilation modes for weaning; however, no study has presented a head-to-head comparison of the efficacy of different modes for liberation from mechanical ventilation. Therefore, we conducted this network meta-analysis to assess the relative efficacy of each technique with the aim of providing treatment recommendations to physicians in daily clinical practice.

## Methods

We performed this systematic review and network meta-analysis using established guidelines from the Preferred Reporting Items for Systematic Reviews and Meta-Analyses for Network Meta-Analyses (PRISMA-NMA) (17, 18) (Supplementary Table 1). The review protocol was registered in the Open Science Framework (OSF, protocol available at https://osf.io/fs8ze).

### Data Sources and Search Strategy

We performed a comprehensive search without language restrictions using PubMed, Embase, and China National Knowledge Infrastructure (https://www.cnki.net) from inception until April 01, 2020. The goal was to identify all relevant trials while screening the titles and reviewing the abstracts. To ensure that no randomized controlled trials were missing, gray literature (conference abstracts and doctoral theses) were searched, and the reference lists of included articles were reviewed. Further ongoing trials were searched using Google Scholar, and the US Government Clinical Trials Database (www.ClinicalTrials.gov). The search terms comprised “Ventilation Weaning,” “T-piece,” “Pressure Support Ventilation,” “Synchronized Intermittent Mandatory Ventilation,” “Automatic Tube Compensation,” “Continuous Positive Airway Pressure,” “Adaptive Support Ventilation,” “Neurally Adjusted Ventilatory Assist,” “Proportional Assisted Ventilation,” and “SmartCare,” along with a list of all interventions and possibly relevant key words (Supplementary Table 2).

### Study Selection

We included randomized control studies on mechanically ventilated adults (at least 18 years of age) that reported at least one of extubation associated outcomes (weaning success or failure, proportion requiring re-intubation, or mortality) with respiratory failure of various etiologies and received invasive mechanical ventilation (MV) for at least 24 h. The comparison included two or more ventilation modes for weaning. We excluded trials that evaluated neonatal or pediatrics subjects, enrolled extubated patients directly to non-invasive ventilation for weaning, compared without controls or same ventilation mode but different parameters.

Two authors (HJJ, LJOY) independently selected trials that met the inclusion criteria, and another author (PHC) adjudicated differences. In the case of disagreement, the same authors consulted with another author (CHL) to obtain decisions after group discussion.

### Data Extraction and Bias Assessment

Two reviewers (HJJ and PHC) independently assessed the eligibility of identified citations and extracted data. Data extraction was performed with a form to capture information regarding study, participants, and treatment characteristics. We contacted the corresponding authors for missing data (Supplementary Table 3).

The same authors independently appraised each study using the Cochrane Risk of Bias (RoB) tool (Supplementary Figure 1) (19). We produced RoB graphs using the software Review Manager 5.3 (20). Discrepancies were resolved by consensus in consultation with a third reviewer (CHL) or deliberation through group discussion.

### Outcome Measures

1. Weaning success: the absence of the requirement for invasive mechanical ventilation support, without cardiac arrest events, or mortality for 48 h after the extubation (translaryngeal tube) or withdrawal (tracheostomy tube), or as defined by the study authors (Supplementary Table 4).
2. The proportion requiring reintubation: the patient requiring reintubation in 48 h after extubation or as defined by the study authors.
3. All-cause mortality: hospital mortality or as defined by the study authors.

### Data Synthesis and Statistical Analysis

We performed the network meta-analysis using a frequentist approach and provided a point estimated using a 95% confidence interval (CI) with the frequency distribution. All network meta-analyses were done with the statistical package “netmeta” in R 3.4.2 (R Core Team, Vienna, Austria) and Stata version 16 (Stata Corp, College Station, Texas, USA). We examined the symmetry and geometry of the evidence by producing a network plot with nodes for the number of study subjects and connection size corresponding to the number of studies. The estimation of mixed estimate of the network summary effects was calculated using the combination of the direct and indirect treatment effect and comprised network structure (Supplementary Figure 2) (21). For the dichotomous variables, we produced the pooled odds ratio (OR) with 95% CIs to summarize the effects of each comparison tested using a random-effects model (22), allowed for across studies variation.

The probability of a mode being ranked was calculated as its surface under the cumulative ranking curve (SUCRA) in frequentist framework, which is the percentage of efficacy achieved by an approach compared with an imaginary approach that is always the best without uncertainty (i.e., SUCRA = 100%). SUCRA provides a hierarchy of treatments and accounts for the variance of all relative intervention effects (23–25). In the frequentist model, *P*-score is an interpretation analogous to the SUCRA and measures certainty of whether a treatment is better than another treatment. Higher *P*-score scores correspond to a greater weaning success rate, lower proportion requiring reintubation and lower mortality (26).

Forest plots summarized relative mean effects, 95% CIs, and *P*-score for all comparisons together (27). The *P*-score results were summarized in a rank-heat plot (28). We used a multivariate random-effects meta-regression with a consistency model by White et al. (29). We assessed potential inconsistencies by comparing deviance and deviance estimates for each comparison between consistency and inconsistency using a random-effects design-by-treatment interaction model (30, 31) and the node-splitting technique (32, 33). Statistical significance was set at 5% for both analyses.

Network transitivity was examined by visually inspecting tables with study-related characteristics that may modify treatment effects, including differences in patient characteristics, study designs, details of the intervention, and differences in measurements of the outcome. Sensitivity analyses were performed to examine the validity of study findings (34). Subgroup analyses were conducted based on the following effect modifiers: endotracheal prosthesis defined as the methods for attaching to a ventilator such as ventilation through endotracheal tube or tracheostomy, publication year before and after 2008 which was the first published randomized control trial of PAV for weaning, and patients with COPD.

We evaluated whether treatment effects for the outcomes were robust and examined the relationship using random-effect network meta-regression with study characteristics. Comparison-adjusted funnel plots and Egger's test were also used to assess publication bias or other small study effects for available interventions (23). The quality of evidence derived from the GRADE framework. (35, 36) (Supplementary Table 5).

## Results

### Systematic Literature Review

**Figure 1:**
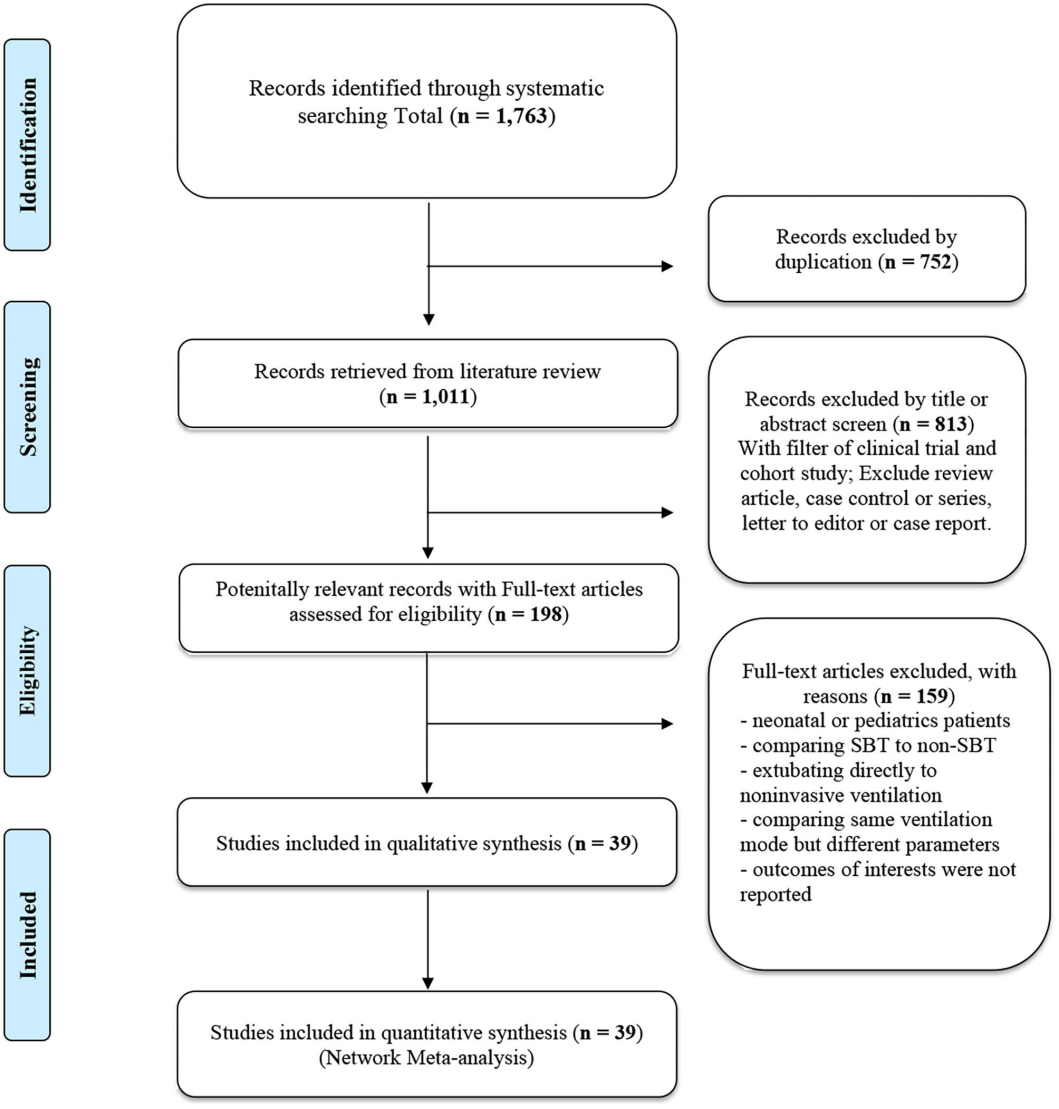
PRISMA flow diagram of included studies.

Totally, 39 articles met our inclusion criteria in our study. The studies regarding neurally adjusted ventilatory assist were excluded because of no adequate information. Figure 1 showed the flowchart. The 39 trials (37–75) investigated a total of 5,953 participants who were randomized into the following interventions: adaptive support ventilation (ASV), automatic tube compensation (ATC), continuous positive airway pressure (CPAP), PAV (including PAV plus mode), pressure support ventilation (PSV), SmartCare, synchronized intermittent mandatory ventilation (SIMV), and the T-piece.

Study characteristics was summarized in Supplementary Table 3. The studies were with sample sizes ranging from 23 to 1,153 patients. There were 62.8% males. The mean age of subjects was 62.1 years old (standard deviation (SD): 8.0 years old), and the mean mechanical ventilation duration prior to randomization was 5.4 days (SD: 3.2 days). The mean Acute Physiology and Chronic Health Evaluation II score was 19.8 (SD: 5.6).

### Result of Weaning Success

**Figure 2:**
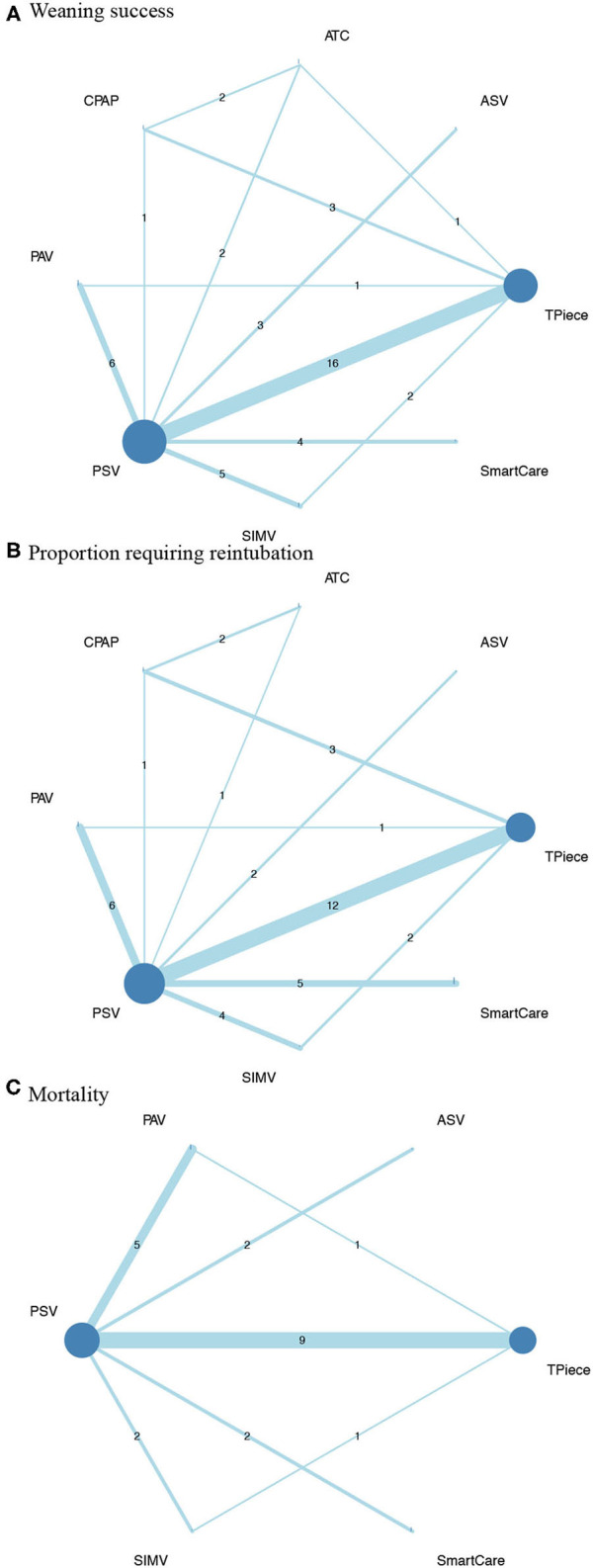
Network plot of comparisons in **(A)**, weaning success **(B)**, proportion requiring reintubation, and mortality among different ventilator modes **(C)**. ASV, Adaptive support ventilation; ATC, Automatic tube compensation; CPAP, Continuous positive airway pressure; PAV, Proportional assist ventilation; PSV, Pressure support ventilation; SIMV, Synchronized intermittent mandatory ventilation.

**Figure 3:**
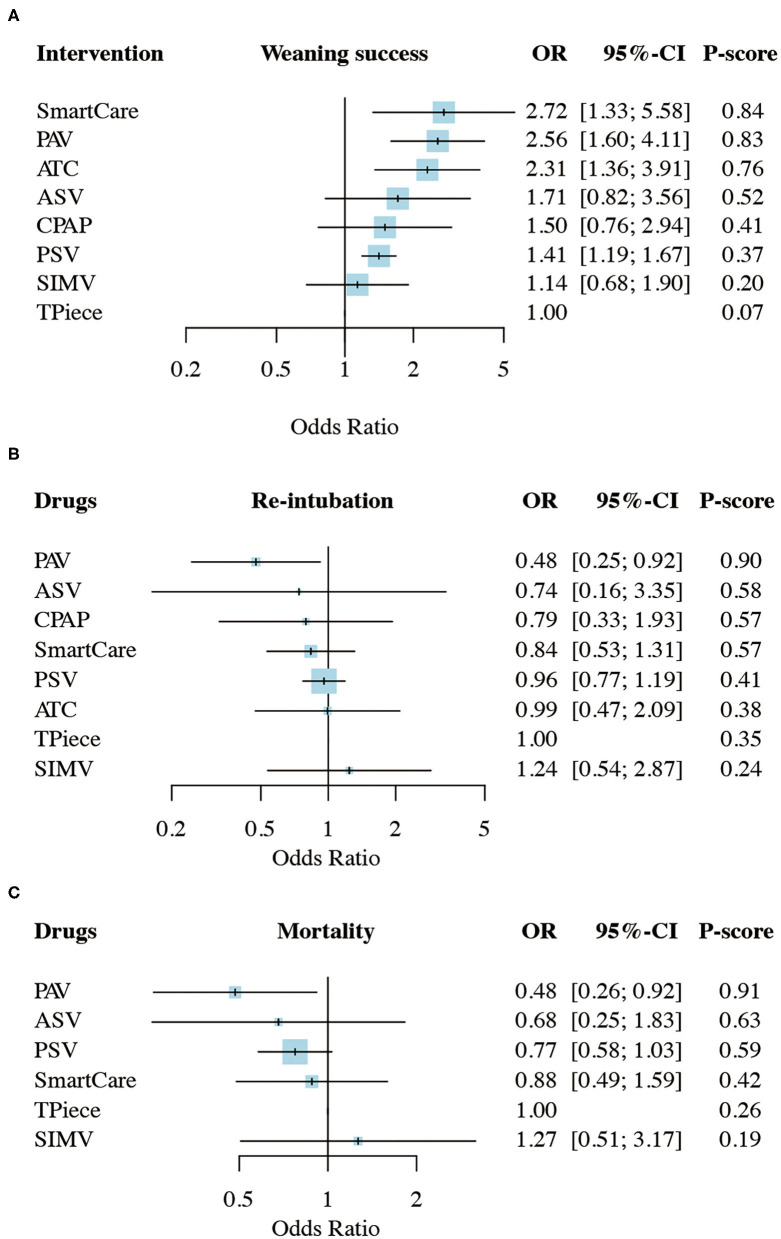
Network meta-analysis results of **(A)**, weaning success **(B)** proportion requiring reintubation, and mortality **(C)**. ASV, Adaptive support ventilation; ATC, Automatic tube compensation; CPAP, Continuous positive airway pressure; PAV, Proportional assist ventilation; PSV, Pressure support ventilation; SIMV, Synchronized intermittent mandatory ventilation; OR, odds ratio; CI, confidence interval.

**Figure 4:**
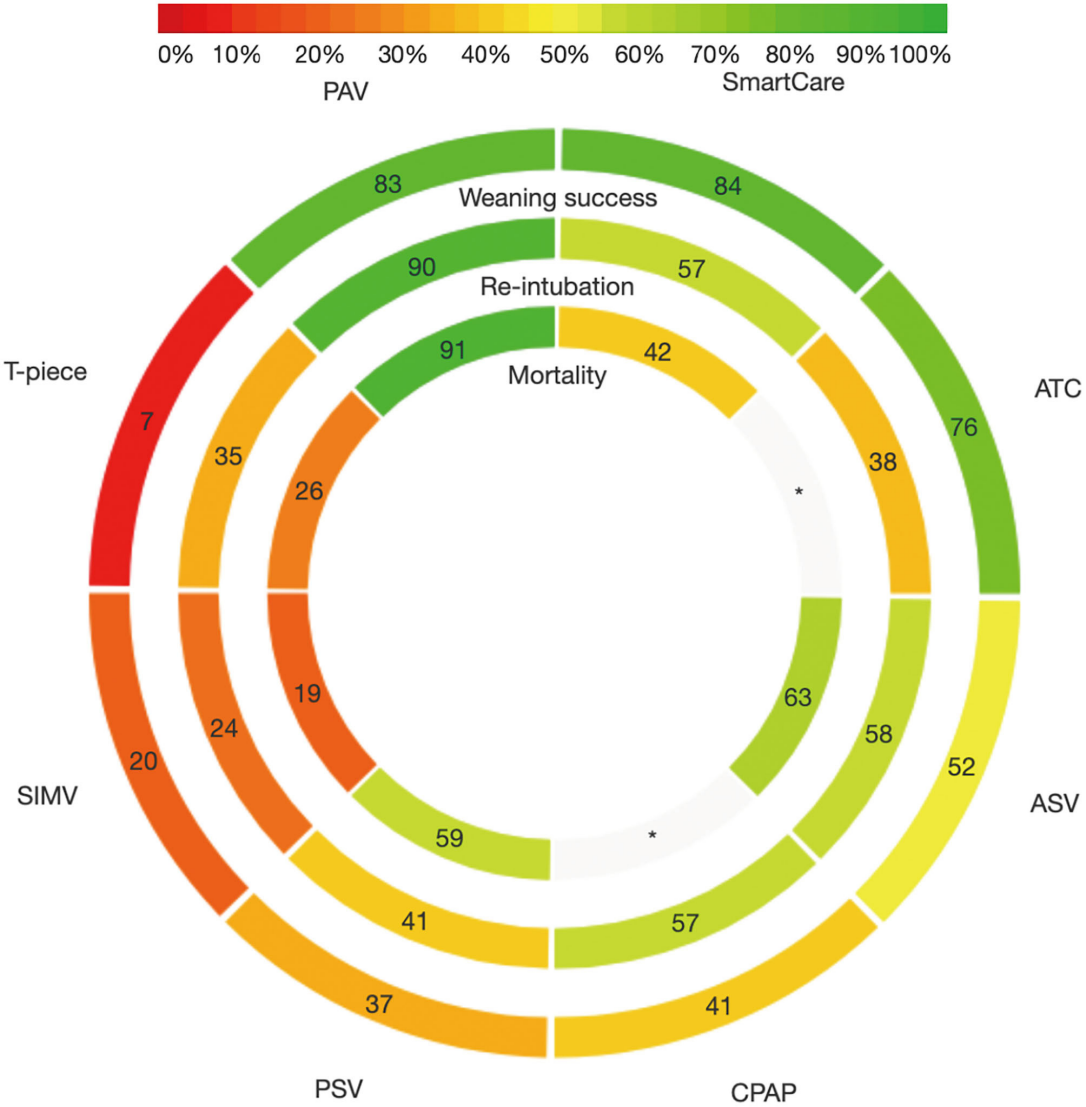
Rank-heat plot of *P*-score values among different ventilator modes targeting outcomes of weaning success, proportion requiring reintubation and mortality. ASV, Adaptive support ventilation; ATC, Automatic tube compensation; CPAP, Continuous positive airway pressure; PAV, Proportional assist ventilation; PSV, Pressure support ventilation; SIMV, Synchronized intermittent mandatory ventilation; OR, odds ratio; CI, confidence interval.

There were 36 studies (5,008 patients; 8 treatment nodes) regarding the weaning success with maintained transitivity (Figure 2A). Figure 3A presented the results of weaning success, in which the T-piece was used as a comparator. PAV and SmartCare had a significantly better weaning success rate (PAV: OR, 2.56; 95% CI, 1.60–4.11, P-score: 0.83; SmartCare: OR, 2.72; 95% CI, 1.33–5.58, P-score: 0.84; Figure 4). Supplementary Table 6 shows details of the head-to-head comparison of outcomes.

In the sensitivity analyses of, the *P*-score rankings were changed. PAV became the first ranking in weaning success after omitting the small trials (<25th percentiles) or excluding the high risk-of-bias studies (Supplementary Figure 4). All the subgroup analyses revealed similar results, favoring PAV, including patients with an endotracheal prosthesis type of translaryngeal tube (PAV: OR, 3.12; 95% CI, 1.67–5.82; *P*-score: 0.91) and patients with COPD (PAV: OR, 5.89; 95% CI, 1.31–26.43; P-score: 0.88). In the subgroup of publication years after 2008, PAV and SmartCare had similar efficacy for weaning success (PAV: OR, 2.69; 95% CI, 1.66–4.37, P-score: 0.84; SmartCare: OR, 2.91; 95% CI, 1.29–6.54, P-score: 0.85; Supplementary Figure 4).

### Result of Proportion Requiring Reintubation

There were 31 studies (4,644 patients; 8 treatment nodes) regarding the proportion requiring reintubation with maintained transitivity (Figure 2B). Figure 3B presented the results of the proportion requiring reintubation, in which the T-piece was used as a comparator. PAV had a significantly lower proportion of re-intubation (PAV: OR, 0.48; 95% CI, 0.25–0.92; *P*-score: 0.90) (Figure 4). The first ranking of PAV was unchanged in sensitivity analyses. Moreover, PAV was the most highly ranked intervention in all subgroups (Supplementary Figure 4).

### Result of Mortality

There were 18 studies (3,727 patients; 6 treatment nodes) regarding mortality with maintained transitivity (Figure 2C). Figure 3C presented the results of mortality rate, in which the T-piece was used as a comparator. PAV was significantly beneficial for mortality (PAV: OR, 0.48; 95% CI, 0.26–0.92; P-score: 0.91) (Figure 4). The top ranking of PAV was unchanged in sensitivity analyses. Moreover, PAV was the most highly ranked intervention in all subgroups, but no statistical significance (Supplementary Figure 4).

### Inconsistency, Meta-Regression Analysis, and Publication Bias

In the design-by-treatment interaction model, there was no evidence of global inconsistency in any outcomes (Supplementary Table 7). In the node-splitting model, there was no evidence of substantial statistical inconsistency between direct and indirect evidence except for the proportion requiring reintubation. There was local inconsistency between the comparisons of ATC vs. CPAP, ATC vs. PSV, and CPAP vs. the T-piece.

In the meta-regression analysis, there was no relationship between the intervention outcomes and the study characteristics (Supplementary Table 8). In all the outcomes, there was no evidence of potential small-study effects or publication bias according to Egger's test and the comparison-adjusted funnel plots, respectively (Supplementary Figure 5).

## Discussion

We conducted a systematic review and network meta-analysis to compare the efficacy of different modes for weaning in patients with mechanical ventilation. PAV and SmartCare had a higher ratio for weaning success. Furthermore, PAV ranked as the best intervention for the lowest proportion requiring reintubation and mortality rate comparing mechanical ventilator with any other modes for weaning. Therefore, PAV seemed to be the best weaning mode in our analysis.

The difficulty of weaning was associated with two major parameters: the duration of the weaning and the level of support pressure (76–78). In concerned with latest study (65), patients in the 30-min PSV had a higher rate of weaning success and lower hospital mortality than patients in the 2-h T-piece SBT. There was no difference of the proportion requiring reintubation and tracheotomy rate between these two groups. Similarly, the comparison between PSV and T-piece in our study, PSV increased the rate of weaning success but did not reduce the rates of reintubation and mortality. The T-piece seemed to be more difficult than the PSV because there was no ventilation support for the T-piece.

Weaning can be accomplished by several methods. PSV or T-piece as a period of SBTs remain common methods for weaning. Automated modes of mechanical ventilation achieve synchrony of interaction between patient and ventilator, thereby improving the patient–ventilator relationship with closed-loop control system. ASV is an automated system that adapts inspiratory pressure to achieve a target tidal volume and a desired minimum minute ventilation. SmartCare measures selected respiratory variables, adapts ventilator output by an explicit algorithm and automates the conduct of SBTs. PAV automatically adjusts the flow assist and volume assist to represent constant fractions of the measured values resistance and elasticity of the patient's respiratory system instantaneously (13, 14).

Patient–ventilator asynchrony was seen in ~25–80% of patients with mechanical ventilation and might result in patient distress, prolonged mechanical ventilation, and weaning failure (79, 80). PAV delivered positive pressure ventilation in proportion to instantaneous inspiratory effort, improved patient–ventilator synchrony, and unloaded the respiratory muscles without the risk of over-assistance and periodic breathing (81). In a pilot study, Bosma et al. (55) demonstrated that the weaning protocols of PAV were not inferior to PSV regarding utility, safety, and feasibility. Based on its advantages, PAV might improve quality of life and decreased health care costs (82). In our subgroup analysis of publication years after 2008 when the PAV mode was first applied to weaning, we found that PAV was associated with a higher rate of successful weaning and a lower rate of reintubation, but there was no significant difference in mortality.

COPD was a disease with increasing prevalence and mortality worldwide (83). In severe conditions, mechanical ventilation was used to maintain adequate oxygenation and reduce the work of breathing. In previous studies (84, 85), patients with COPD had a longer weaning phase and a lower success rate of the weaning procedures compared to patients without COPD. However, Elganady et al. (60) showed that PAV was less patient–ventilator asynchrony, reduced period of mechanical ventilation, and shortened ICU and hospital stays. In our study, we found that weaning with PAV in patients with COPD was associated with a higher rate of weaning success.

Despite limited real-world experience about weaning with PAV, our results have demonstrated promising efficacy and a higher weaning success, a lower reintubation rate, and lower mortality than any other ventilation mode. However, due to a paucity of a variety of weaning methods in comparison with PAV, optimization of the weaning strategy was required in further studies.

The strength of this review was that we simultaneously compared seven different ventilation modes for weaning in patients with mechanical ventilation in ICUs using a network meta-analysis. To avoid bias, a comprehensive search, study selection, data extraction, and bias assessment by two reviewers were performed. We produced a rank-heat plot to summarize the results and allowed readers to quickly visualize the highest ranked choice. Besides, inconsistency was properly identified by the node-splitting and design-by-treatment model. Finally, the certainty of evidence was rated by the GRADE approach.

There were several limitations in our study. Firstly, patient population were various cross the studies and it was difficult to separate the individual studies into subgroup analysis to conduct network meta-analysis with more specific aspect. Secondly, the variety of ventilation setting prior to or during weaning might flaw the clinical efficacy; therefore, we summarized the characteristics of included studies in visually inspecting tables to provide more detail information. Lastly, due to the small number of included studies, the results should be interpreted with caution. Despite these limitations, we still hoped our findings provided a rationale for designing future large-scale randomized control trials.

## Conclusion

According to our network meta-analysis, weaning with PAV and SmartCare results in a higher rate of weaning success. Furthermore, PAV reduce reintubation rate and mortality in comparison with other methods of weaning. We hope that this evidence about the benefits and risks when choosing weaning methods for weaning will help physicians to properly provide the optimal course of actions for patients. However, the further head-to-head randomized control trials are warranted to examine the effects of different ventilation modes for weaning.

## Data Availability Statement

The original contributions presented in the study are included in the article/Supplementary Material, further inquiries can be directed to the corresponding author/s.

## Author Contributions

H-JJ and L-JO-Y conceptualized the research goals, planned the analyses, and guided the literature review. H-JJ and P-HC extracted the data from the included studies. P-HC, CL, and C-HL participated in processing the data and doing the statistical analysis. H-JJ and P-HC wrote the first draft of the paper. S-ET and C-HL reviewed and edit. All authors read and approved the final manuscript.

## Funding

Financial support of this study was from Tri-Service General Hospital/National Defense Medical Centre (Nos. TSGH-D-110141 and TSGH-E-110199).

## Conflict of Interest

The authors declare that the research was conducted in the absence of any commercial or financial relationships that could be construed as a potential conflict of interest.

## Publisher's Note

All claims expressed in this article are solely those of the authors and do not necessarily represent those of their affiliated organizations, or those of the publisher, the editors and the reviewers. Any product that may be evaluated in this article, or claim that may be made by its manufacturer, is not guaranteed or endorsed by the publisher.
