# Supplementary material for: Methods of Weaning From Mechanical Ventilation in Adult: A Network Meta-Analysis
Source: Front Med (Lausanne). 2021 Oct 4;8:752984. doi: 10.3389/fmed.2021.752984 (PMC8521009; doi:10.3389/fmed.2021.752984)
Supplement: Supplementary file 1 [file Data_Sheet_1.docx]

**Supplementary Data File 1**

**Methods of Weaning from Mechanical Ventilation in Adult: A Network Meta-analysis**

Hong-Jie Jhou MD, Po-Huang Chen MD, Liang-Jun Ou-Yang MD, Chin Lin PhD, Shih-En Tang MD, Cho-Hao Lee MD

**Contents**

Table S1. PRISMA for Network Meta-Analyses

Table S2. Search strategy

Table S3. Characteristics of included studies

Table S4. Definition of weaning success

Table S5. GRADE approach for rating the quality of treatment effect estimate

Table S6. Results the head-to-head comparison of network meta-analysis

Table S7. Estimation of inconsistency

Table S8. Meta-regression analyses

Table S9. Reference list of included studies

Figure S1. Risk of bias table of included studies

Figure S2. Direct-indirect evidence contribution plot

Figure S3. Comparison direct and indirect evidence in network meta-analysis

Figure S4. Subgroup analyses and sensitivity analyses

Figure S5. Comparison-adjusted funnel plots and Egger’s test

**Table S1. PRISMA for Network Meta-Analyses**

| **Section/**  **Topic** | **Item** | **Checklist Item** | **Reported on Page #** |
| --- | --- | --- | --- |
| **TITLE** | | | |
| Title | 1 | Identify the report as a systematic review *incorporating a network meta-analysis (or related form of meta-analysis).* | 1-2 |
| **ABSTRACT** | | | |
| Structured summary | 2 | Provide a structured summary including, as applicable:  **Background:** main objectives  **Methods:** data sources; study eligibility criteria, participants, and interventions; study appraisal; and *synthesis methods, such as network meta-analysis.*  **Results:** number of studies and participants identified; summary estimates with corresponding confidence/credible intervals; *treatment rankings may also be discussed. Authors may choose to summarize pairwise comparisons against a chosen treatment included in their analyses for brevity.*  **Discussion/Conclusions:** limitations; conclusions and implications of findings.  **Other:** primary source of funding; systematic review registration number with registry name. | 3 |
| **INTRODUCTION** | | | |
| Rationale | 3 | Describe the rationale for the review in the context of what is already known*, including mention of why a network meta-analysis has been conducted.* | 4-5 |
| Objectives | 4 | Provide an explicit statement of questions being addressed, with reference to participants, interventions, comparisons, outcomes, and study design (PICOS). | 5 |
| **METHODS** |  |  |  |
| Protocol and registration | 5 | Indicate whether a review protocol exists and if and where it can be accessed (e.g., Web address); and, if available, provide registration information, including registration number. | 5 |
| Eligibility criteria | 6 | Specify study characteristics (e.g., PICOS, length of follow-up) and report characteristics (e.g., years considered, language, publication status) used as criteria for eligibility, giving rationale. *Clearly describe eligible treatments included in the treatment network, and note whether any have been clustered or merged into the same node (with justification).* | 5-6 |
| Information sources | 7 | Describe all information sources (e.g., databases with dates of coverage, contact with study authors to identify additional studies) in the search and date last searched. | 6 |
| Search | 8 | Present full electronic search strategy for at least one database, including any limits used, such that it could be repeated. | 5-6,  Table S2 |
| Study selection | 9 | State the process for selecting studies (i.e., screening, eligibility, included in systematic review, and, if applicable, included in the meta-analysis). | 6 |
| Data collection process | 10 | Describe method of data extraction from reports (e.g., piloted forms, independently, in duplicate) and any processes for obtaining and confirming data from investigators. | 6-7 |
| Data items | 11 | List and define all variables for which data were sought (e.g., PICOS, funding sources) and any assumptions and simplifications made. | 7 |
| Geometry of the network | S1 | Describe methods used to explore the geometry of the treatment network under study and potential biases related to it. This should include how the evidence base has been graphically summarized for presentation, and what characteristics were compiled and used to describe the evidence base to readers. | 7-9 |
| Risk of bias within individual studies | 12 | Describe methods used for assessing risk of bias of individual studies (including specification of whether this was done at the study or outcome level), and how this information is to be used in any data synthesis. | 7-9 |
| Summary measures | 13 | State the principal summary measures (e.g., risk ratio, difference in means). *Also describe the use of additional summary measures assessed, such as treatment rankings and surface under the cumulative ranking curve (SUCRA) values, as well as modified approaches used to present summary findings from meta-analyses.* | 7-9 |
| Planned methods of analysis | 14 | Describe the methods of handling data and combining results of studies for each network meta-analysis. This should include, but not be limited to:  *Handling of multi-arm trials;*  *Selection of variance structure;*  *Selection of prior distributions in Bayesian analyses; and Assessment of model fit.* | 7-9 |
| Assessment of Inconsistency | S2 | Describe the statistical methods used to evaluate the agreement of direct and indirect evidence in the treatment network(s) studied. Describe efforts taken to address its presence when found. | 7-9 |
| Risk of bias across studies | 15 | Specify any assessment of risk of bias that may affect the cumulative evidence (e.g., publication bias, selective reporting within studies). | 7-9 |
| Additional analyses | 16 | Describe methods of additional analyses if done, indicating which were pre-specified. This may include, but not be limited to, the following:  Sensitivity or subgroup analyses;  Meta-regression analyses;  *Alternative formulations of the treatment network; and Use of alternative prior distributions for Bayesian analyses (if applicable).* | 7-9 |
| **RESULTS†** |  |  |  |
| Study selection | 17 | Give numbers of studies screened, assessed for eligibility, and included in the review, with reasons for exclusions at each stage, ideally with a flow diagram. | 9,  Figure 1 |
| Presentation of network structure | S3 | Provide a network graph of the included studies to enable visualization of the geometry of the treatment network. | Figure 2 |
| Summary of network geometry | S4 | Provide a brief overview of characteristics of the treatment network. This may include commentary on the abundance of trials and randomized patients for the different interventions and pairwise comparisons in the network, gaps of evidence in the treatment network, and potential biases reflected by the network structure. | 9-11, Table S3 |
| Study characteristics | 18 | For each study, present characteristics for which data were extracted (e.g., study size, PICOS, follow-up period) and provide the citations. | 9-11, Table S3 |
| Risk of bias within studies | 19 | Present data on risk of bias of each study and, if available, any outcome level assessment. | Figure S1 |
| Results of individual studies | 20 | For all outcomes considered (benefits or harms), present, for each study: 1) simple summary data for each intervention group, and 2) effect estimates and confidence intervals. *Modified approaches may be needed to deal with information from larger networks.* | 10-11 |
| Synthesis of results | 21 | Present results of each meta-analysis done, including confidence/credible intervals. *In larger networks, authors may focus on comparisons versus a particular comparator (e.g. placebo or standard care), with full findings presented in an appendix. League tables and forest plots may be considered to summarize pairwise comparisons.* If additional summary measures were explored (such as treatment rankings), these should also be presented. | 10-11, Table S5, Figure S3 |
| Exploration for inconsistency | S5 | Describe results from investigations of inconsistency. This may include such information as measures of model fit to compare consistency and inconsistency models, *P* values from statistical tests, or summary of inconsistency estimates from different parts of the treatment network. | 10-11, Table S7 |
| Risk of bias across studies | 22 | Present results of any assessment of risk of bias across studies for the evidence base being studied. | 10-11 |
| Results of additional analyses | 23 | Give results of additional analyses, if done (e.g., sensitivity or subgroup analyses, meta-regression analyses*, alternative network geometries studied, alternative choice of prior distributions for Bayesian analyses,* and so forth). | 10-11 |
| **DISCUSSION** | | | |
| Summary of evidence | 24 | Summarize the main findings, including the strength of evidence for each main outcome; consider their relevance to key groups (e.g., healthcare providers, users, and policy-makers). | 11 |
| Limitations | 25 | Discuss limitations at study and outcome level (e.g., risk of bias), and at review level (e.g., incomplete retrieval of identified research, reporting bias). *Comment on the validity of the assumptions, such as transitivity and consistency. Comment on any concerns regarding network geometry (e.g., avoidance of certain comparisons).* | 13 |
| Conclusions | 26 | Provide a general interpretation of the results in the context of other evidence, and implications for future research. | 13-14 |
| **FUNDING** | | | |
| Funding | 27 | Describe sources of funding for the systematic review and other support (e.g., supply of data); role of funders for the systematic review. This should also include information regarding whether funding has been received from manufacturers of treatments in the network and/or whether some of the authors are content experts with professional conflicts of interest that could affect use of treatments in the network. | 14 |

PICOS = population, intervention, comparators, outcomes, study design.

* Text in italics indicateS wording specific to reporting of network meta-analyses that has been added to guidance from the PRISMA statement.

**Table S2. Search Strategy**

| **Database** | **Search Detail** |
| --- | --- |
| **Pubmed & Embase** | Search **(((((("nava"[All Fields] OR "neurally adjusted ventilatory assist"[All Fields] OR "neurally adjusted ventilator assist"[All Fields]))) OR (“Adaptive Support Ventilation” [All Fields] OR “ASV” [All Fields]) OR (“Continuous Positive Airway Pressure” [All Fields] OR “CPAP”[All Fields]) OR (“Automatic Tube Compensation” [All Fields] OR “ATC”) OR “Synchronized Intermittent Mandatory Ventilation” [All Fields] OR “SIMV”) OR ("Pressure Support Ventilation"[All Fields] OR “PSV” [All Fields]) OR ((“T-piece” OR “t tube”))) OR (“Proportional Assist Ventilation” [All Fields] OR “PAV” [All Fields]) OR “SmartCare” OR “NeoGanesh”) AND ((("ventilation"[MeSH Terms] OR "ventilation"[All Fields] OR "respiration"[MeSH Terms] OR "respiration"[All Fields]) AND ("weaning"[MeSH Terms] OR "weaning"[All Fields])))) AND ((groups[tiab] OR trial[tiab] OR randomly[tiab] OR randomized[tiab] OR controlled clinical trial[pt] OR randomized controlled trial[pt]))** |
| **CNKI** | 撤机 (Ventilation Weaning)  导管阻力补偿 (ATC)  同步间隙指令通气 (SIMV)  适应性支持通气 (ASV)  压力支持通气 (PSV)  比例辅助通气 (PAV)  持续气道正压 (CPAP)  T管 (T-piece)  自主呼吸试验 (SBT)  智能监护脱机模式 (Smartcare) |

**Table S3. Characteristics of included studies**

| **Author year; Design (Country)** | **Population** | **Age**  **(mean)** | **Male (%)** | **Mean MV** | **Mean PS** | **COPD (%)** | **Mode prior to Intervention** | **Intervention 1** | **Intervention 2** | **Intervention 3** |
| --- | --- | --- | --- | --- | --- | --- | --- | --- | --- | --- |
| Brochard 1994;  RCT (Italy) | 109 MICU patients with MV for more than 24 hours who  failed the 2h of T-tube trial | 59.1 yrs | 67.8 | 14 days | SAPS: 12 | 28 | NR | T- piece for a maximum of 2 h | PSV of 8 cmH2O and PEEP of 4cmH2O | SIMV |
| Esteban 1995;  RCT (Spain) | 130 ICU patients with MV for more than 24 hours who  failed initial SBT | 58.0 yrs | NR | 9.3 days | APACHE II: 19.5 | 30 | ACV | ACV to IMV;  IMV: initial rate was 10.0 (±2.2) breaths/minute, decrease the ventilator rate by 2-4/mins twice a day | ACV to PSV; PSV: Pressure support ventilation of 5 cmH2O | ACV to T-piece: T-piece circuit for up to two hours each day |
| Esteban 1997;  RCT (Spain) | 484 ICU patients with MV for at least 48 hours | 64.0 yrs | 70.9 | 6.0 days | SAPS II: 36 | 21 | ACV | ACV to T-piece; T- piece for a maximum of 2 h | ACV to PSV PSV: Pressure support ventilation of 7 cmH2O and PEEP ≤ 5 cmH20 | None |
| Koh 2000;  RCT (Korea) | 36 MICU patients with MV for more than 72 hours | 61.0 yrs | 63.9 | 12 days | APACHE II: 35.0 | 0 | PSV | PSV to T-piece; T- piece (additional 1 hour period of T-piece trial) | PSV: Pressure support ventilation of ±7.6 cm H2O for 30 minutes | None |
|  |  |  |  |  |  |  |  |  |  |  |
| Sulzer 2001;  RCT (Switzerland) | 36 post-CABG patients for MV | 59.5 yrs | 72.2 | 0.1 days | NR | NR | ASV/SIMV | ASV to ASV | SIMV to PSV; PSV: Pressure support ventilation of 5 cmH2O | None |
| Vitacca 2001;  RCT (Italy) | 52 tracheostomized and COPD patients with MV more than 15 days who  failed the T-tube trial | 72.5 yrs | NR | >15 days | APACHE II: 17.0 | 100 | PSV | PSV to T-piece; T- piece for duration from 30 min to 8h (30, 60 min, 2, 4, 8 h) | PSV: Pressure support ventilation: the pressure was decreased by 2 cmH2O twice a day until 8 cmH2O for 8 hours | None |
| Haberthur 2002; RCT (Switzerland) | 90 MICU patients with MV for more than 24 hours | 57.0 yrs | 64.4 | 6.3 days | APACHE II: 20.0 | 10 | NR | T- piece for a maximum of 2 h | PSV: Pressure support of 5 cmH2O and PEEP of 5 cmH2O | ATC: Automatic tube compensation, PEEP of 5 cmH2O |
| Koksal 2004;  RCT (Turkey) | 60 ICU patients with MV for more than 48 hours | 74.0 yrs | 46.7 | NR | APACHE II: 18.5 | NR | NR | T-piece (4L/min) for 2hours | PSV: Pressure support ventilation 10 cmH2O and PEEP of 5 cmH2O | CPAP: PEEP 5 cmH2O |
| Matic 2004;  RCT (Croatia) | 260 MICU patients with MV for more than 48 hours | 51.2 yrs | 65.4 | 6.8 days | APACHE II: 25.0 | 13 | NR | T- piece for a maximum of 2 h | PSV: Pressure support ventilation of 8 cmH20 and PEEP ≤ 5cmH20 | None |
| Ma 2005;  RCT (China) | 55 SICU postoperative patients with MV for more than 48 hours | 53.6 yrs | 76.4 | 1.5 days | NR | NR | NR | T-piece (< 5L/min) for 30-120min | PSV: Pressure support of 7 cmH2O for 30-120min | None |
| Jiang 2006;  RCT (China) | 38 COPD patients receiving MV for at least 3 days | 83.3 yrs | NR | NR | NR | 100 | NR | SmartCare | PSV | None |
|  |  |  |  |  |  |  |  |  |  |  |
|  |  |  |  |  |  |  |  |  |  |  |
| Cohen 2006;  RCT (Israel) | 99 ICU patients with MV for more than 24 hours | 63.3 yrs | 52.5 | 6.6 days | APACHE II: 17.5 | NR | PSV | PSV to ATC; ATC: ventilator circuit with flow-triggering (2 L/min) and CPAP of 5 cm H2O, with inspiratory ATC set at 100% | PSV to CPAP; CPAP: ventilator circuit with flow triggering (2 L/min) and CPAP of 5 cm H2O | None |
| Matic 2007;  RCT (Croatia) | 136 MICU patients with COPD receiving MV for more than 24 hours | 58.0 yrs | 57.1 | 5.0 days | APACHE II: 30.0 | 100 | NR | T- piece for a maximum of 2 h | Pressure support ventilation of 5 cmH20 | None |
| Rose 2008;  RCT (Australia) | 102 patients receiving MV for greater than 24 h | 52.5 yrs | 56.7 | 5.2 days | APACHE II: 17.5 | NR | VCV/PCV | SmartCare | VCV/PCV to PSV; PSV | None |
| Xirouchaki 2008;  RCT (Greece) | 208 ICU patients with MV for more than 36 hours | 60.9 yrs | 66.3 | 4.0 days | APACHE II: 15.5 | NR | VCV/PCV | PAV+: the initial percentage of assist was set to 60-80% | VCV/PCV to PSV: PSV: the inspiratory pressure was set to 20-25 cmH2O (including PEEPE) | None |
| Cohen 2009;  RCT (Israel) | 180 ICU patients with MV for more than 24 hours | 64.2 yrs | 63.9 | 6.1 days | APACHE II: 20.6 | NR | NR | ATC: patients breathed through the ventilatory circuit using flow-triggering and PEEP of 5 cmH2O, FiO2 less than 0.5 with the addition of ATC 100% | PSV: patients breathed through the ventilatory circuit using flow-triggering and PEEP of 5 cmH2O, FiO2 less than 50% with the addition of 7 cmH2O of pressure support) | None |
| Deng 2009;  RCT (China) | 187 ICU patients with MV | 53.0 yrs | 56.5 | NR | NR | 36 | NR | T-piece: FiO2 40% for 30 mins | PSV: Pressure 5 cmH2O, PEEP 5 cmH2O for 30 mins | None |
| Ma 2010;  RCT (China) | 62 patients with MV | NR | NR | 2.0 days | NR | NR | NR | SmartCare | SIMV + PSV | None |
| Figueroa 2010;  RCT (US) | 118 ICU patients with MV for more than 24 hours | 51.2 yrs | 89.3 | NR | APACHE II: 19.5 | NR | NR | SIMV: Fsimv:12-14 times/min, PEEP<5 cmH2O, FiO2<45% with gradually reduced Fsimv by 2 times/min every 6 hrs | PSV: Pressure 16-18 cmH2O with gradually reduced pressure by 2-4 cmH2O every 6 hrs | None |
| Molina-Saldarriaga 2010;  RCT (Colombia) | 50 MICU patients with COPD receiving MV for more than 48 hours | 63.7 yrs | 46.0 | 6.0 days | APACHE II: 14.8 | 100 | NR | T-piece for 30 mins | CPAP: 85% intrinsic PEEP for 30 mins | None |
| HE 2010;  RCT (China) | 120 ICU patients with MV for more than 24 hours | 52.0 yrs | 56.7 | 2.5 days | APACHE II: 21.3 | 48.3 | NR | T-piece for 30-120min | PSV: Pressure support of 5-7 cmH2O for 30-120min | None |
| Li 2010;  RCT (China) | 32 MICU patients with COPD receiving MV for more than 72 hours | 65.7 yrs | 68.8 | 5.0 days | SAPS II: 37.8 | 100 | SIMV + PSV | SIMV + PSV to PSV; PSV Pressure 8-10 cm H2O for 2-4 hr | SIMV + PSV to SIMV; SIMV: initially as breathing rate for 2-4 hrs with gradually reducing 1-2 times of the guaranteed breaths/minute until 4 breaths/minute every single day | None |
| Kirakli 2011;  RCT (Turkey) | 97 ICU patients with COPD receiving MV | 64.5 yrs | 91.8 | 2.4 days | APACHE II: 16.0 | 100 | AC(VC) | ASV: minute ventilation 100 ml/kgIBW, 50 after 1 h and 30 after 2 h, followed by 2h SBT | AC (VC) to PSV;  PSV: Pressure 15 cmH2O with 2 cmH2O interval decrease and the goal of 7 cmH2O, PEEP 3-5 cmH2O | None |
|  |  |  |  |  |  |  |  |  |  |  |
| Cekmen 2011;  RCT (Turkey) | 40 ICU patients with MV for more than 48 hours | 69.0 yrs | 55 | 6.7 days | APACHE II: 28.5 | NR | SIMV | SIMV to T-piece; T-piece 4 L/min | SIMV to CPAP; CPAP: PEEP ≦5 cmH2O | None |
| Chen 2012;  RCT (China) | 30 ICU patients with COPD receiving MV | 65.8 yrs | 60 | 10.2 days | NR | 100 | SIMV + PSV | SIMV + PSV to PSV; PSV: Pressure support of 5-8 cmH2O for 2-4 hr | SIMV + PSV to SIMV; SIMV: initially as breathing rate for 2-4 hrs with gradually reducing 1-2 times of the guaranteed breaths/minute until 4 breaths/minute | None |
| Schädler 2012;  RCT (Germany) | 300 postoperative patients receiving MV for more than 9 hours | 68.0 yrs | 69.7 | 0.2 days | APACHE II: 16 | 13.7 | NR | SmartCare | PSV | None |
| Burns 2013;  RCT (Canada) | 92 ICU patients receiving MV at least 24 hours | 63.8 yrs | 58.7 | 5.5 days | APACHE II: 23.7 | 25 | NR | SmartCare | PSV; Pressure 5-7 cmH2O or pressure 10-12 cmH2O ± PEEP 5 cmH2O for 30-120 mins | None |
| Sasikumar 2013;  RCT (India) | 23 ICU patients with MV for more than 48 hours | 48.6 yrs | 69.6 | NR | APACHE II: 20.7 | NR | ACV/SIMV | PAV+: starting at 100% assist, reduced by 10–20% every 2 h | ACV/SIMV to PSV; PSV: pressure support level required to deliver a tidal volume of at least 7 mL/kg for 30 min | None |
| Tong 2014;  RCT (China) | 36 COPD patients with MV | 75.3 yrs | NR | NR | NR | 100 | NR | SmartCare | PSV |  |
| Zhang 2014;  RCT (China) | 208 ICU patients with MV | 71.3 yrs | 56.3 | 4.7 days | NR | 36.0 | NR | T-piece with an oxygenation setting of 4 L/min (30%). | PSV: Pressure 5 cmH2O, PEEP 5 cmH2O, FiO2 30% for 30 mins | None |
| Elganady 2014;  RCT (Egypt) | 60 MICU patients with COPD receiving MV for more than 24 hours | 59.7 yrs | 81.7 | NR | NR | 100 | AC(VC) | PAV+: starting at 70% assist, reduced by 10–20% every 2 h | AC(VC) to PSV; PSV: Pressure 5–8 cm H2O for 120 mins | None |
|  |  |  |  |  |  |  |  |  |  |  |
|  |  |  |  |  |  |  |  |  |  |  |
|  |  |  |  |  |  |  |  |  |  |  |
| Mohamed 2014;  RCT (Egypt) | 50 MICU patients with MV | 65.2 yrs | 72.0 | 5.7 days | APACHE II: 14.8 | 100 | ACV | ASV: Minute volume was decreased to 30% in the ASV mode to achieve lower pressure support levels, for 2-h period | ACV to PSV; PSV: Pressure support with initial level 15 cmH2O and gradually decreased to 7 cmH2O by 2 cmH2O, for 2-h period | None |
| Teixeira 2015;  RCT (Brazil) | 160 ICU patients with MV for more than 24 hours | 44.5 yrs | 65.6 | 6.6 days | APACHE II: 22.7 | 22.5 | VCV | VCV to T-piece;  T-piece with supplemental oxygen to maintain SpO2 >92% | VCV to PSV; PSV: Pressure support with 7 cmH2O | PAV+: adjusted percentage support to maintain subjects in a comfortable range (0.3– 0.7 J/L) |
| Taniguchi 2015;  RCT (Brazil) | 70 ICU patients with MV for more than 24 hour | 64.0 yrs | 55.7 | NR | NR | NR | NR | SmartCare | PSV;  Pressure 5–7 cmH2O and PEEP 5 cmH2O | None |
| Bosma 2016;  RCT (Canada) | 50 ICU patients with MV for more than 36 hours | 64.8 yrs | 50.0 | 5.8 days | APACHE II: 26.5 | 20.0 | ACV/PSV | PAV+: PAV of 10–20% gain | ACV/PSV to PSV; PSV: Pressure support with 5-6 cmH2O, PEEP of 5 cmH2O | None |
| Pellegrini 2018;  RCT (Brazil) | 190 ICU patients with COPD receiving MV for more than 48 hours | 67.7 yrs | 53.7 | 9.1 days | SAPS III: 76.5 | 100 | NR | T-piece, continuously supplied humidified oxygen to achieve a saturation of at least 92% in the absence of positive pressure, for 30 mins | PSV: Pressure support of 10 cmH2O with adjusted PEEP and FiO2 to achieve SaO2 at least 92% for 30 mins | None |
| Botha 2018;  RCT (Australia) | 50 ICU patients with MV for more than 24 hours | 63.2 yrs | 59.2 | 3.4 days | APACHE III: 76.7 SASP: 45.5 | NR | NR | PAV+: 70% support and weaned to 30% support by decrements of 10% as tolerated | PSV: Start with pressure support level required and weaned to 10 cmH2O as tolerated | None |
| Chittawatanarat 2018;  RCT (Thailand) | 520 SICU postoperative patients with MV for at least 12 hours | 56.0 yrs | 26.7 | 1.2 days | APACHE II: 11.0 | 7.9 | NR | T-piece, with an oxygenation setting of 10-15 L/min | PSV: inspiratory pressure 5- 7 cmH2O, PEEP 5 cmH2O, FiO2 0.4, expiration triggered at 25% of peak inspiratory flow rate). | None |
| Subira 2019; RCT (Spain) | 1153 ICU patients with MV for at least 24 hours | 62.2 yrs | 62.9 | 4.0 days | APACHE II: 16.0 | 19.8 | NR | T-piece for 2 hours | PSV: 30-minute with pressure support 8 cmH2O and zero PEEP | None |
| ACV: Assist-control ventilation, AC(VC): Assisted volume-controlled ventilation, APACHE II: Acute Physiology and Chronic Health Evaluation II score, ASV: Adaptive support ventilation, ATC: Automatic tube compensation, COPD: Chronic obstructive pulmonary disease, CPAP: Continuous positive airway pressure, FiO2: Fraction of inspiration O_2_, ICU: Intensive care unit, MICU: Medical intensive care unit, MV: Mechanical ventilator, PAV: Proportional assist ventilation, PCV: Pressure control ventilation, PEEP: Positive end-expiratory pressure, PSV: Pressure support ventilation, RCT: Randomized controlled trial, SICU: Surgical intensive care unit, SIMV: Synchronized intermittent mandatory ventilation, VCV: Volume control ventilation, | | | | | | | | | | |

**Table S4. Definition of weaning success**

| **Author year** | **Definition of weaning success** |
| --- | --- |
| Brochard 1994 | Not defined |
| Esteban 1995 | Not defined |
| Esteban 1997 | No further requirement for ventilation within 48 hours following extubation |
| Koh 2000 | No further requirement for ventilation within 48 hours following extubation |
| Vitacca 2001 | No further requirement for ventilation within 48 hours following extubation |
| Haberthur 2002 | No further requirement for ventilation within 48 hours following extubation |
| Koksal 2004 | No further requirement for ventilation within 48 hours following extubation |
| Matic 2004 | No further requirement for ventilation within 48 hours following extubation |
| Ma 2005 | Not defined |
| Cohen 2006 | No further requirement for ventilation within 48 hours following extubation |
| Matic 2007 | No further requirement for ventilation within 48 hours following extubation |
| Xirouchaki 2008 | No further requirement for ventilation within 48 hours following extubation |
| Cohen 2009 | No further requirement for ventilation within 48 hours following extubation |
| Deng 2009 | No further requirement for ventilation within 72 hours following extubation |
| Figueroa 2010 | No further requirement for ventilation within 48 hours following extubation |
| Molina-Saldarriaga 2010 | Not defined |
| HE 2010 | Not defined |
| Li 2010 | No further requirement for ventilation within 48 hours following extubation |
| Kirakli 2011 | No further need for ventilation for 48 hours after extubation or with a tracheostomy cannula at day 28 |
| Cekmen 2011 | No further requirement for ventilation within 48 hours following extubation |
| Chen 2012 | No further requirement for ventilation within 48 hours following extubation |
| Sasikumar 2013 | No further requirement for ventilation within 48 hours following extubation |
| Zhang 2014 | No further requirement for ventilation within 48 hours following extubation |
| Elganady 2014 | No further requirement for ventilation within 72 hours following extubation |
| Mohamed 2014 | No further requirement for ventilation within 48 hours following extubation |
| Teixeira 2015 | No further requirement for ventilation within 48 hours following extubation |
| Bosma 2016 | No further requirement for ventilation within 48 hours following extubation or need more than 12 hours of non-invasive ventilation per 24-hour period |
| Pellegrini 2018 | No further requirement for ventilation within 48 hours following extubation |
| Botha 2018 | No further requirement for ventilation within 48 hours following extubation |
| Chittawatanarat 2018 | No further requirement for ventilation within 48 hours following extubation |
| Subira 2019 | No further requirement for ventilation within 72 hours following extubation |
| Burns 2013 | No further requirement for ventilation within 48 hours following extubation |
| Schädler, 2012 | Not defined |
| Sulzer, 2001 | Not defined |
| Taniguchi 2015 | No further requirement for ventilation within 48 hours following extubation |
| Rose 2008 | No further need for ventilation for 48 hours after extubation |
| Jiang 2006 | No further requirement for ventilation within 48 hours following extubation |
| Ma 2010 | Not defined |
| Tong 2014 | Not defined |

**Table S5. GRADE approach for rating the quality of treatment effect estimate**

Weaning Success


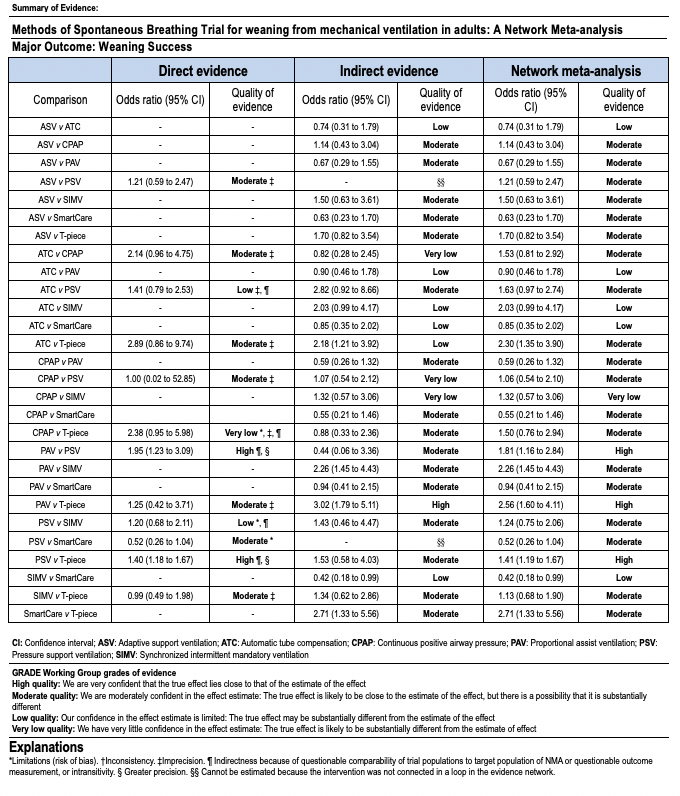


Proportion of reintubation


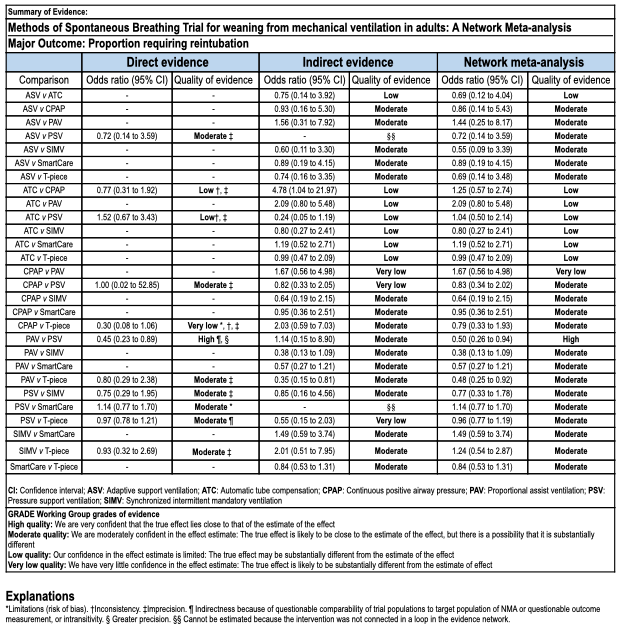


Mortality


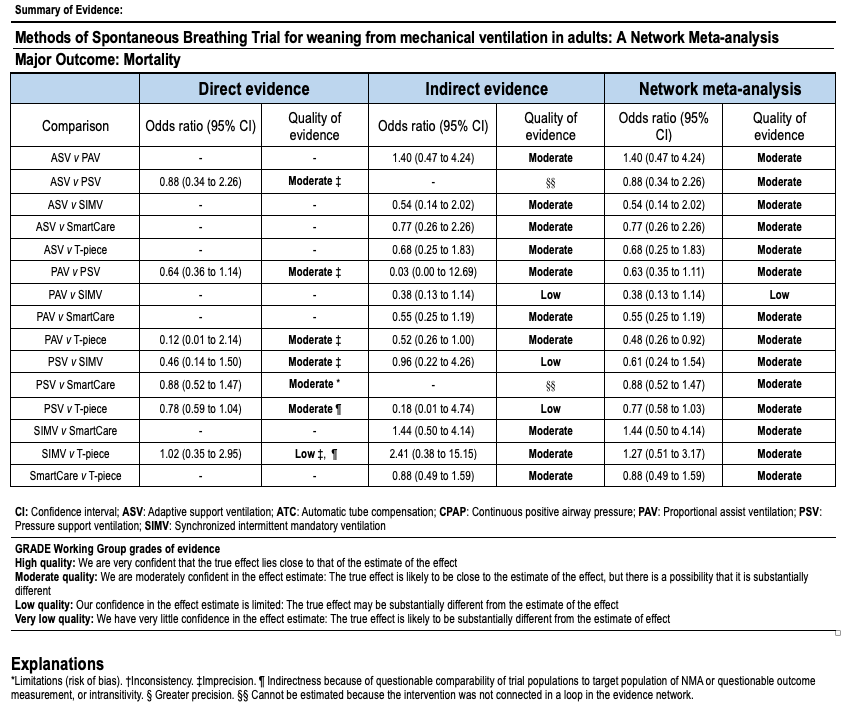


**Table S6. Results of head-to-head comparison of network meta-analysis**

Weaning success: Head-to-head comparison


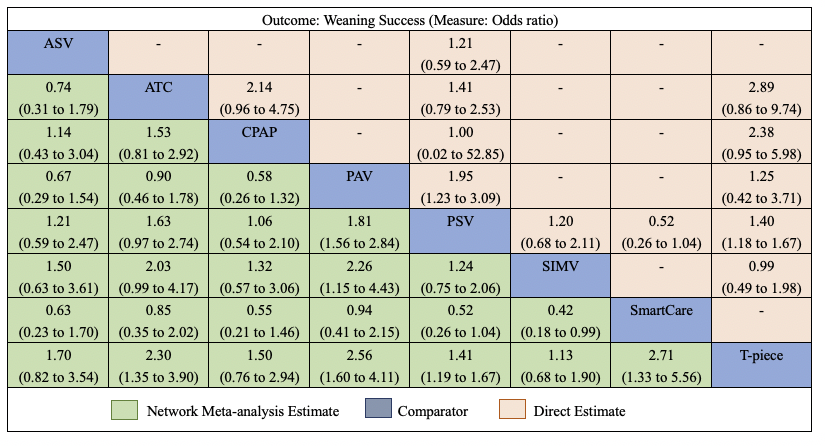


**Table S6.1: Outcomes for weaning success in Network Meta-analysis: Head-to-head comparisons**

Data are presented as the odds ratio with 95% CI in the column-defining treatment compared with the row-defining treatment. Comparisons should be read from left to right.

**ASV**, Adaptive support ventilation; **ATC**, Automatic tube compensation; **CPAP**, Continuous positive airway pressure; **PAV**, Proportional assist ventilation; **PSV**, Pressure support ventilation; **SIMV**, Synchronized intermittent mandatory ventilation; **CI**, Confident interval

Proportion requiring reintubation: Head-to-head comparison


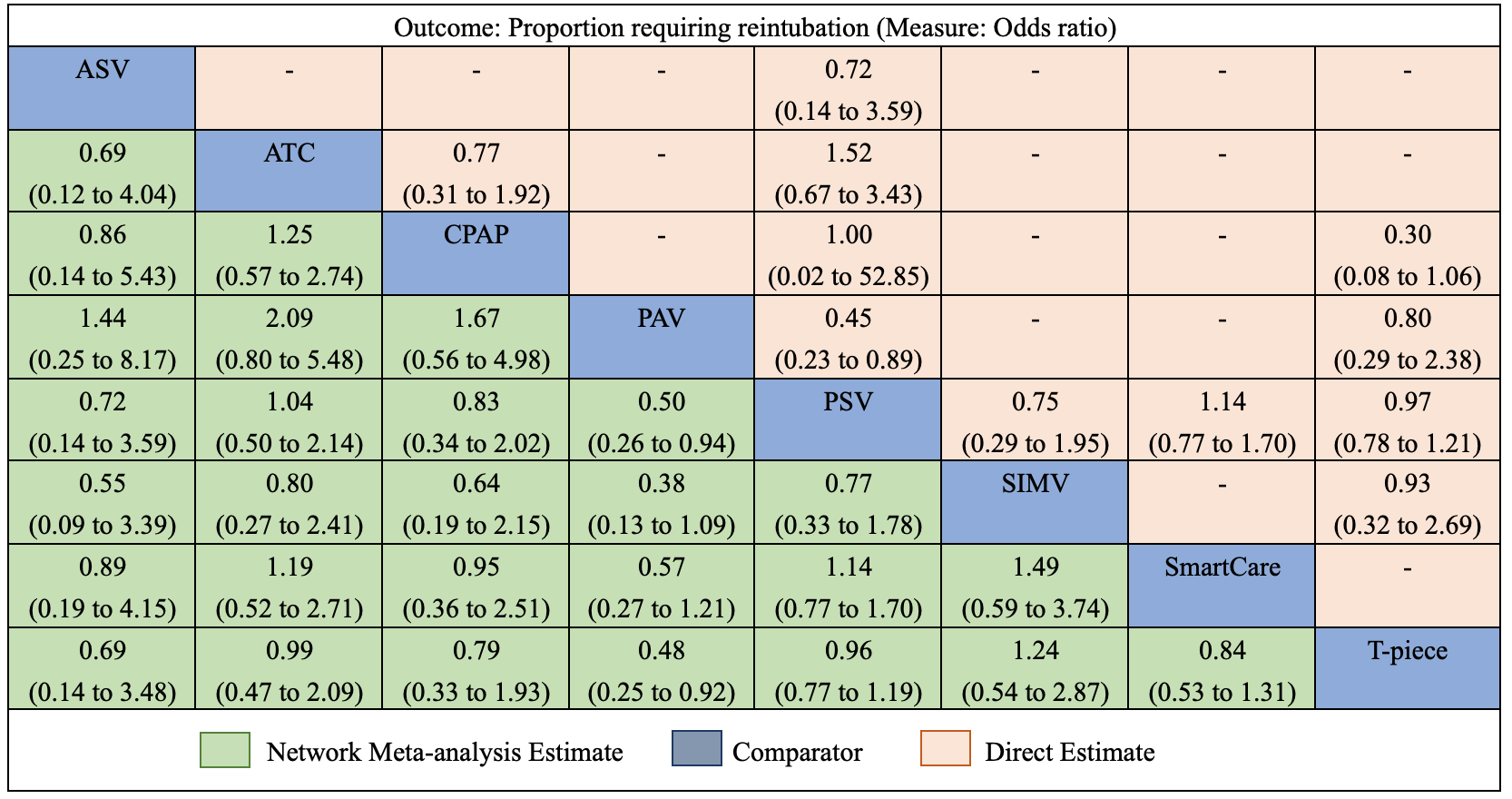


**Table S6.2: Outcomes for proportion requiring reintubation in Network Meta-analysis: Head-to-head comparisons**

Data are presented as the odds ratio with 95% CI in the column-defining treatment compared with the row-defining treatment. Comparisons should be read from left to right.

**ASV**, Adaptive support ventilation; **ATC**, Automatic tube compensation; **CPAP**, Continuous positive airway pressure; **PAV**, Proportional assist ventilation; **PSV**, Pressure support ventilation; **SIMV**, Synchronized intermittent mandatory ventilation; **CI**, Confident interval

Mortality: Head-to-head comparison


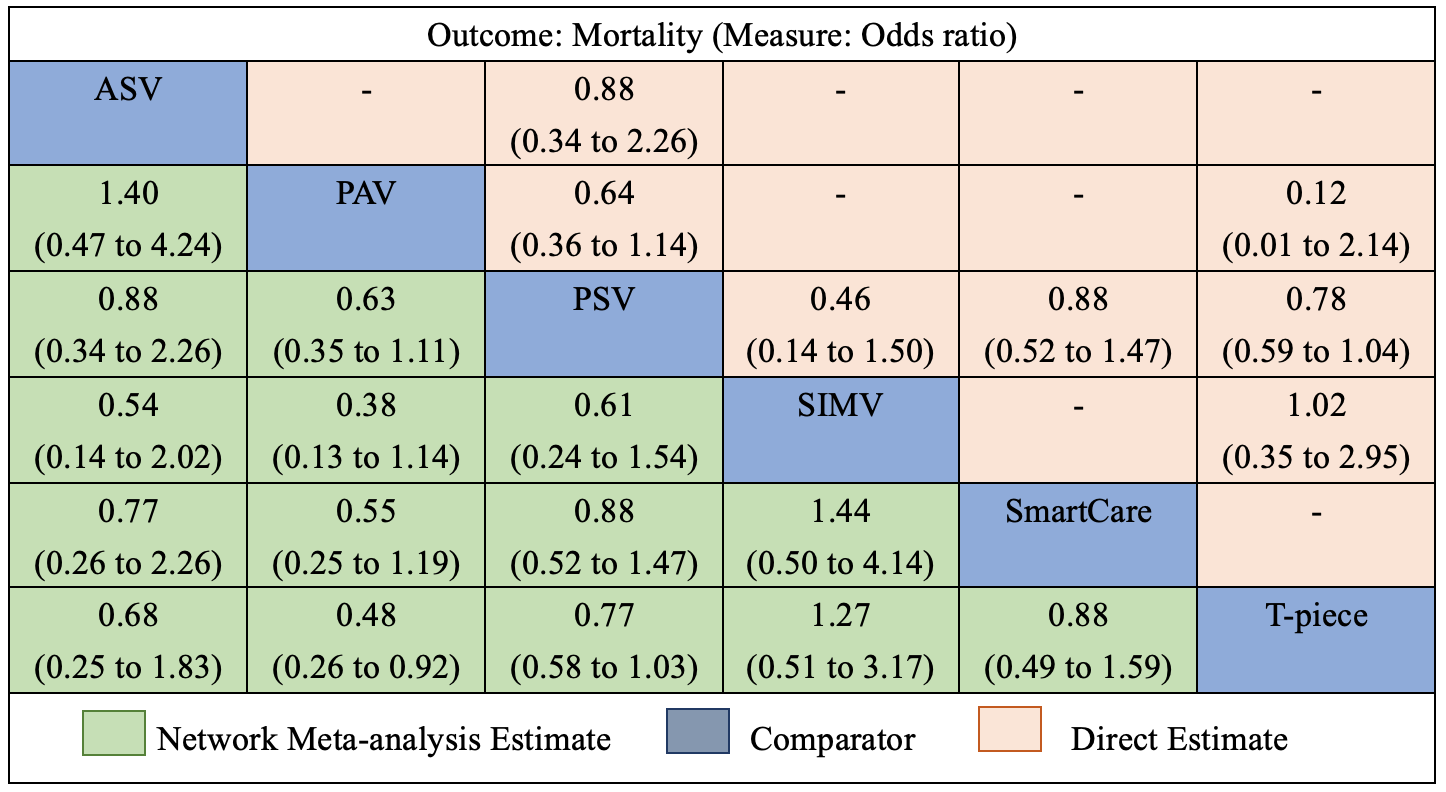


**Table S6.3 Outcomes for mortality Network Meta-analysis: Head-to-head comparisons**

Data are presented as the odds ratio with 95% CI in the column-defining treatment compared with the row-defining treatment. Comparisons should be read from left to right.

**ASV**, Adaptive support ventilation; **ATC**, Automatic tube compensation; **CPAP**, Continuous positive airway pressure; **PAV**, Proportional assist ventilation; **PSV**, Pressure support ventilation; **SIMV**, Synchronized intermittent mandatory ventilation; **CI**, Confident interval

**Table S7. Estimation of inconsistency**

**p-value**: The p-value is set as 0.05. If the p-value is more than 0.05, the null hypothesis cannot be rejected and the consistency assumption could be accepted. Thus, if the p-value is less than 0.05, which means statistically significant that inconsistency exists.

Table S7.1 Estimation of inconsistency in outcome for weaning success

*Design-by-treatment interaction model*

Q statistics to assess homogeneity / consistency

|  | Q | df | p-value |
| --- | --- | --- | --- |
| Total | 31.54 | 34 | 0.5888 |
| Within designs | 21.66 | 25 | 0.6554 |
| Between designs | 9.88 | 9 | 0.3603 |

Design-specific decomposition of within-designs Q statistic

| Design | Q | df | p-value |
| --- | --- | --- | --- |
| ASV:PSV | 4.16 | 2 | 0.1247 |
| ATC:CPAP | 0.53 | 1 | 0.4662 |
| CPAP:T-piece | 0.01 | 1 | 0.9133 |
| PAV:PSV | 3.26 | 4 | 0.5155 |
| PSV:SIMV | 0.85 | 2 | 0.6547 |
| PSV:SmartCare | 2.70 | 3 | 0.4399 |
| PSV:T-piece | 6.37 | 10 | 0.7837 |
| PSV:SIMV:T-piece | 3.78 | 2 | 0.1511 |

Between-designs Q statistic after detaching of single designs

| Design | Q | df | p-value |
| --- | --- | --- | --- |
| ATC:CPAP | 7.98 | 8 | 0.4355 |
| ATC:PSV | 6.25 | 8 | 0.6190 |
| CPAP:T-Piece | 9.16 | 8 | 0.3291 |
| PAV:PSV | 8.68 | 8 | 0.3701 |
| PSV:SIMV | 9.57 | 8 | 0.2964 |
| PSV:T-Piece | 9.44 | 8 | 0.3068 |
| ATC:PSV:T-Piece | 8.10 | 7 | 0.3240 |
| CPAP:PSV:T-Piece | 6.62 | 7 | 0.4698 |
| PAV:PSV:T-Piece | 7.65 | 7 | 0.3641 |
| PSV:SIMV:T-Piece | 9.42 | 7 | 0.2242 |

Q statistic to assess consistency under the assumption of a full design-by-treatment interaction random effects model

|  | Q | df | p-value | tau.within | tau^2^.within |
| --- | --- | --- | --- | --- | --- |
| Between designs | 9.88 | 9 | 0.3603 | 0 | 0 |

*Node-splitting model*

Back-calculation method to split direct and indirect evidence

Fixed effect model:


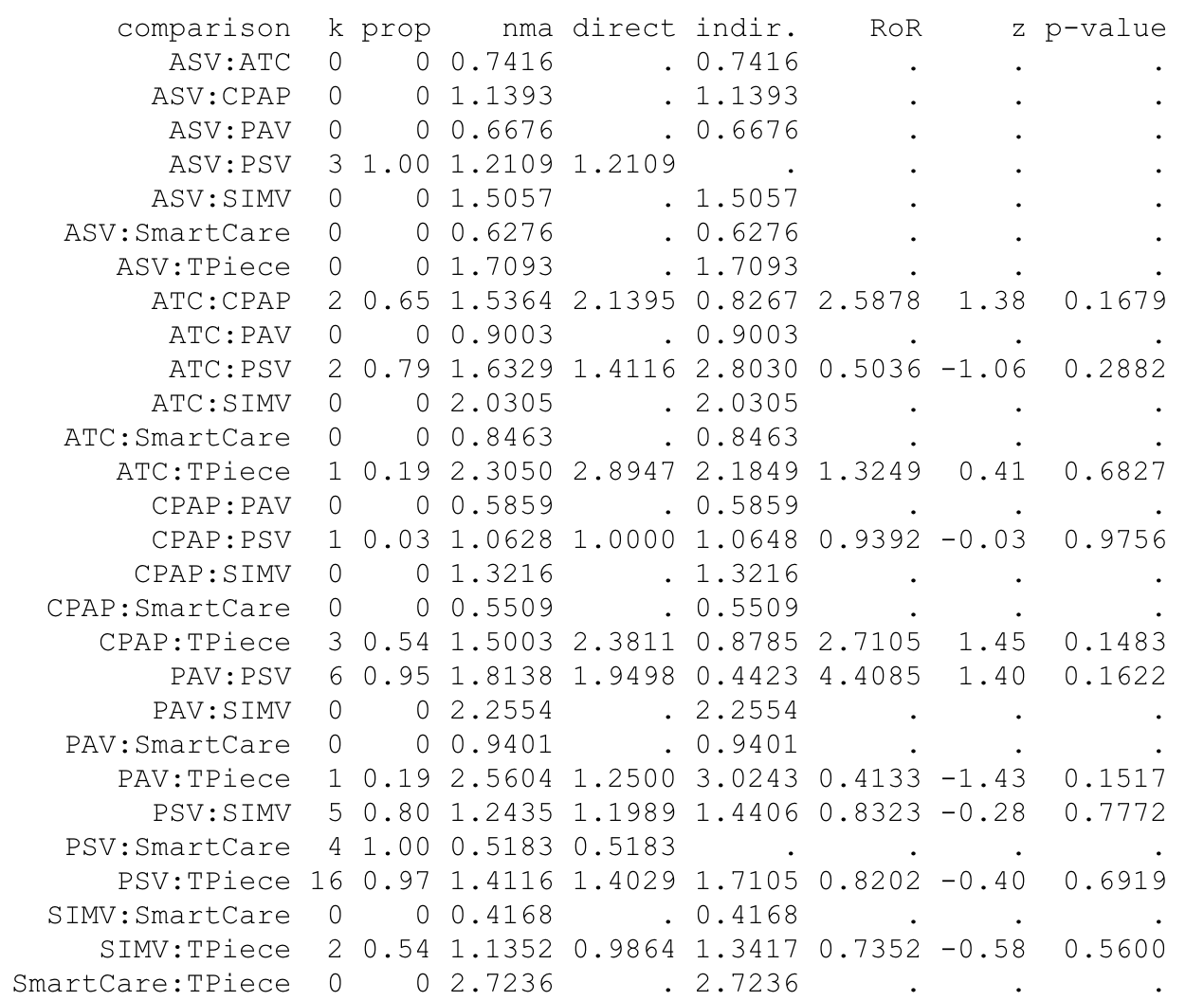


Random effects model:


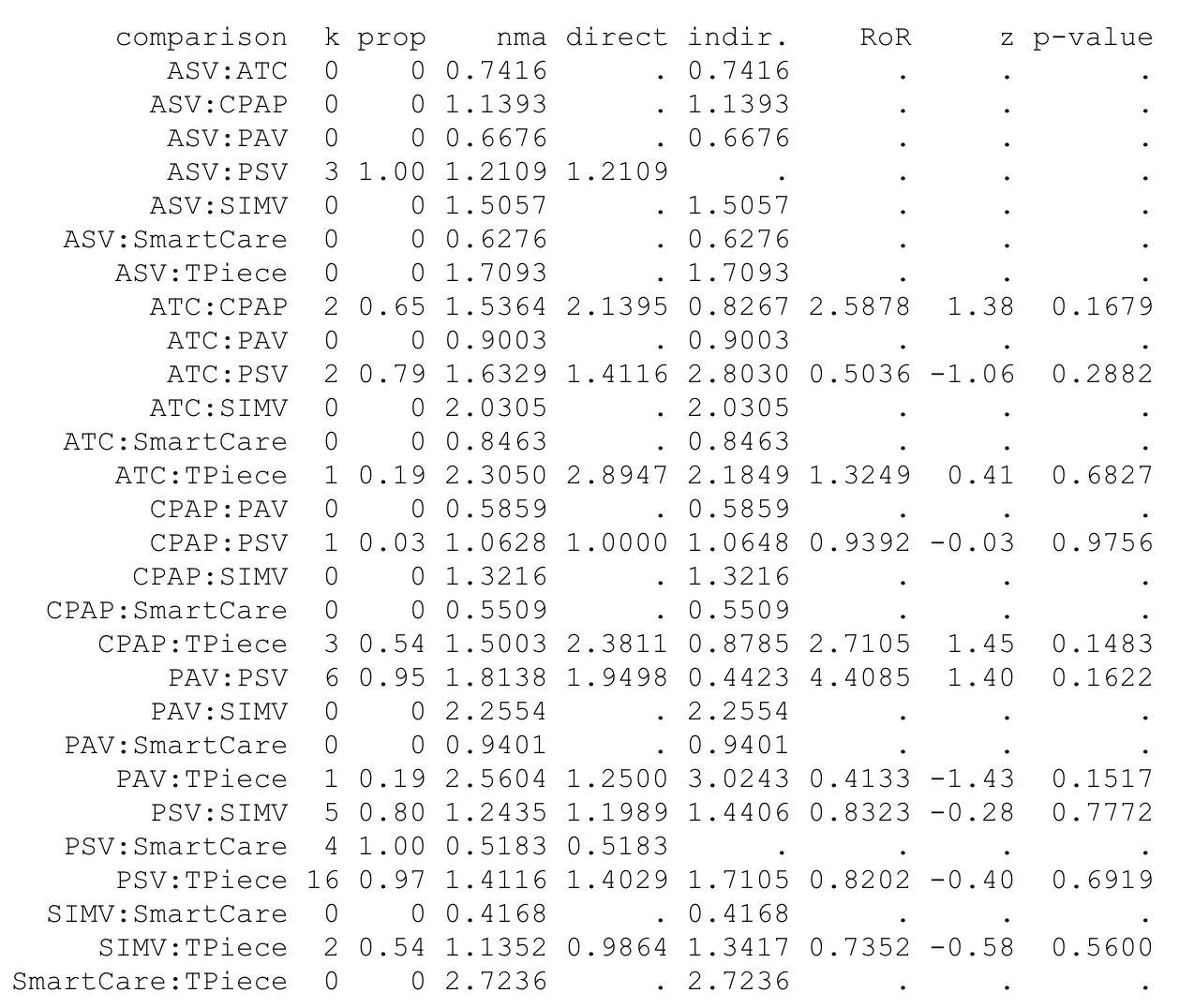


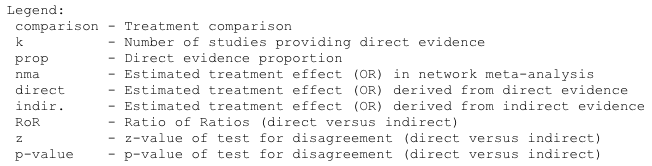


Table S7.2 Estimation of inconsistency in outcome for proportion requiring reintubation

*Design-by-treatment interaction model*

Q statistics to assess homogeneity / consistency

|  | Q | df | p-value |
| --- | --- | --- | --- |
| Total | 21.00 | 28 | 0.8254 |
| Within designs | 12.14 | 21 | 0.9357 |
| Between designs | 8.86 | 7 | 0.2631 |

Design-specific decomposition of within-designs Q statistic

| Design | Q | df | p-value |
| --- | --- | --- | --- |
| ASV:PSV | 0.06 | 1 | 0.8010 |
| ATC:CPAP | 0.80 | 1 | 0.3720 |
| CPAP:TPiece | 0.68 | 1 | 0.4096 |
| PAV:PSV | 1.86 | 4 | 0.7616 |
| PSV:SIMV | 0.00 | 1 | 0.9977 |
| PSV:SmartCare | 1.31 | 4 | 0.8600 |
| PSV:T-piece | 4.98 | 7 | 0.6618 |
| PSV:SIMV:T-piece | 4.98 | 2 | 0.2938 |

Between-designs Q statistic after detaching of single designs

| Design | Q | df | p-value |
| --- | --- | --- | --- |
| ATC:CPAP | 4.80 | 6 | 0.5691 |
| ATC:PSV | 4.80 | 6 | 0.5691 |
| CPAP:T-Piece | 7.19 | 6 | 0.3036 |
| PAV:PSV | 7.62 | 6 | 0.2673 |
| PSV:SIMV | 8.46 | 6 | 0.2063 |
| PSV:T-Piece | 8.22 | 6 | 0.2225 |
| CPAP:PSV:T-Piece | 4.71 | 5 | 0.4527 |
| PAV:PSV:T-Piece | 7.43 | 5 | 0.1905 |
| PSV:SIMV:T-Piece | 8.35 | 5 | 0.1379 |

Q statistic to assess consistency under the assumption of a full design-by-treatment interaction random effects model

|  | Q | df | p-value | tau.within | tau^2^.within |
| --- | --- | --- | --- | --- | --- |
| Between designs | 8.86 | 7 | 0.2631 | 0 | 0 |

*Node-splitting model*

Back-calculation method to split direct and indirect evidence

Fixed effect model:


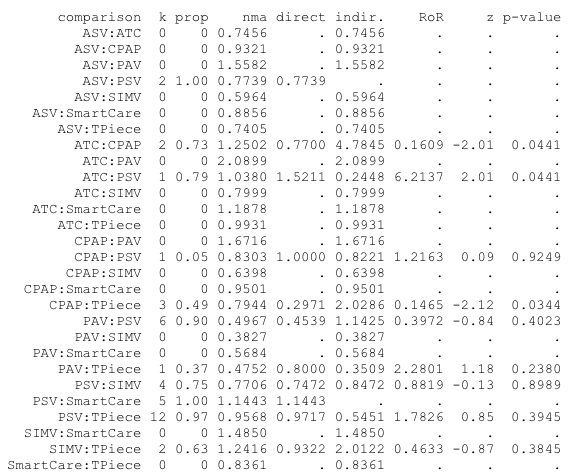


Random effects model:


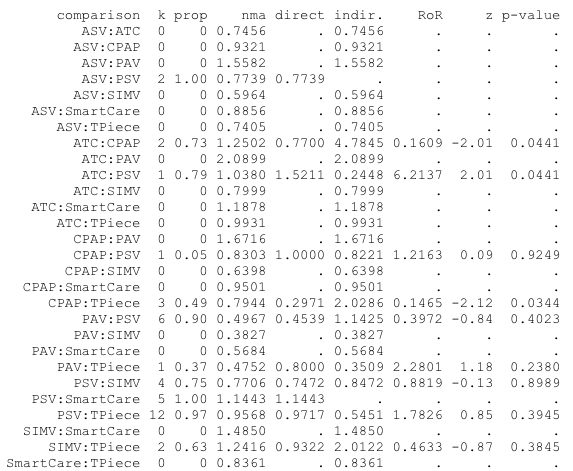


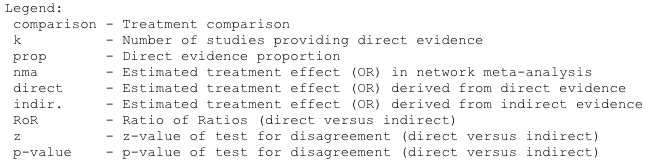


Table S7.3 Estimation of inconsistency in outcome for mortality

*Design-by-treatment interaction model*

Q statistics to assess homogeneity / consistency

|  | Q | df | p-value |
| --- | --- | --- | --- |
| Total | 7.48 | 15 | 0.9429 |
| Within designs | 5.59 | 11 | 0.8994 |
| Between designs | 1.89 | 4 | 0.7552 |

Design-specific decomposition of within-designs Q statistic

| Design | Q | df | p-value |
| --- | --- | --- | --- |
| ASV:PSV | 0.27 | 1 | 0.6023 |
| PAV:PSV | 2.77 | 3 | 0.4277 |
| PSV:SmartCare | 0.42 | 1 | 0.5184 |
| PSV:T-Piece | 2.12 | 6 | 0.9080 |

Between-designs Q statistic after detaching of single designs

| Design | Q | df | p-value |
| --- | --- | --- | --- |
| PAV:PSV | 1.16 | 3 | 0.7623 |
| PSV:SIMV | 1.72 | 3 | 0.6330 |
| PSV:T-Piece | 0.44 | 3 | 0.9325 |
| PAV:PSV:T-Piece | 0.69 | 2 | 0.7070 |
| PSV:SIMV:T-Piece | 1.26 | 2 | 0.5337 |

Q statistic to assess consistency under the assumption of a full design-by-treatment interaction random effects model

|  | Q | df | p-value | tau.within | tau^2^.within |
| --- | --- | --- | --- | --- | --- |
| Between designs | 1.89 | 4 | 0.7552 | 0 | 0 |

*Node-splitting model*

Back-calculation method to split direct and indirect evidence

Fixed effect model:


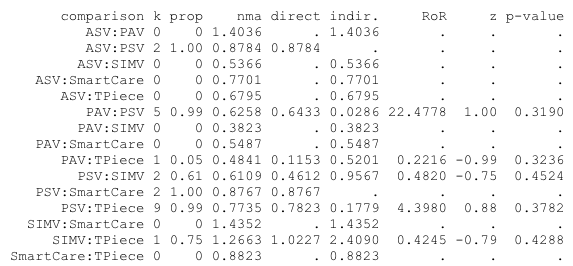


Random effects model:


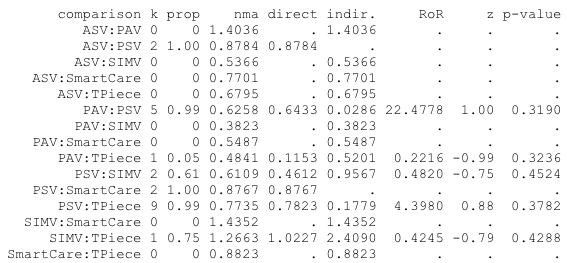


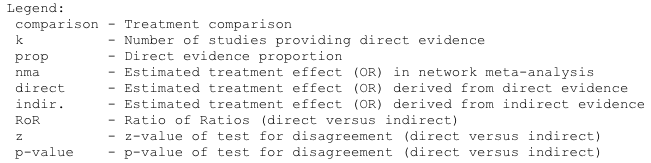


**Table S8. Meta-regression analysis**

Weaning success

*Meta-regression Covariate: Age*

chi2(7) = 6.43

Prob > chi2 = 0.4902


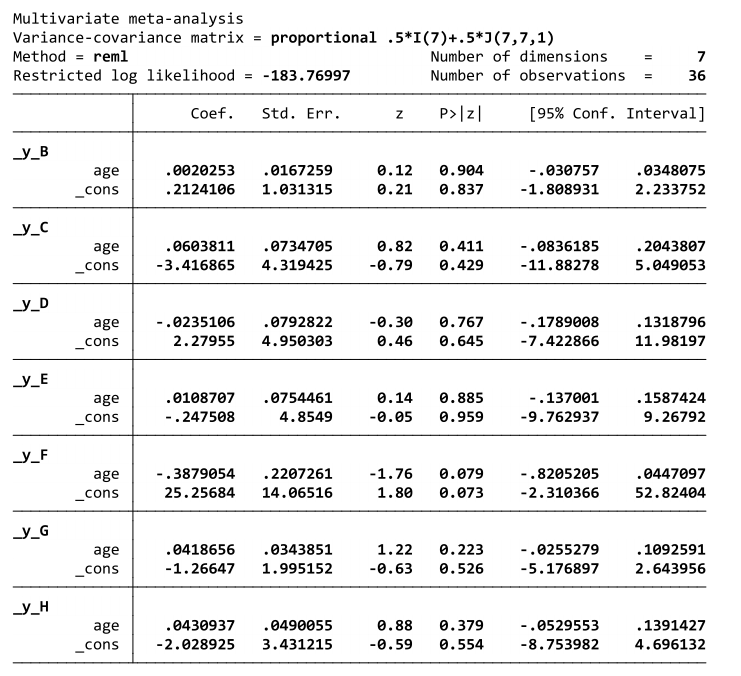


**Table S8.1.1 Meta-regression analysis of covariate as age in outcome for weaning success**

**B**, Pressure support ventilation (PSV); **C,** Synchronized intermittent mandatory ventilation (SIMV); **D**, Automatic tube compensation (ATC); **E**, Continuous positive airway pressure (CPAP); **F**, Adaptive support ventilation(ASV); **G**: Proportional assist ventilation (PAV); **H**: SmartCare

*Meta-regression Covariate: physiology score (APACHE II): insufficient observations*

*Meta-regression Covariate: baseline duration of mechanical ventilation*

chi2(6) = 4.54

Prob > chi2 = 0.6043


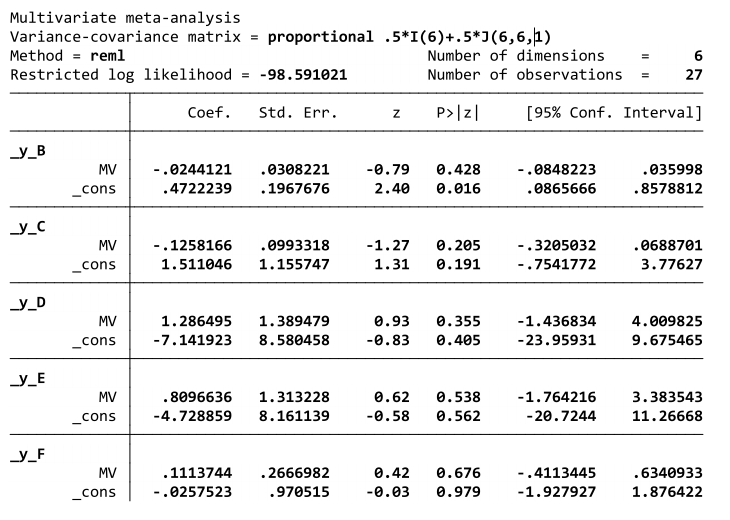

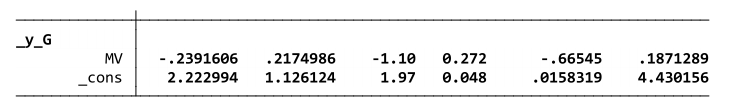


**Table S8.1.2 Meta-regression analysis of covariate as baseline duration of mechanical ventilation in outcome for weaning success**

**B**, Pressure support ventilation (PSV); **C,** Synchronized intermittent mandatory ventilation (SIMV); **D**, Automatic tube compensation (ATC); **E**, Continuous positive airway pressure (CPAP); **F**, Adaptive support ventilation(ASV); **G**: Proportional assist ventilation (PAV)

*Meta-regression Covariate: Definition period of weaning success*

chi2(3) = 3.34

Prob > chi2 = 0.3423


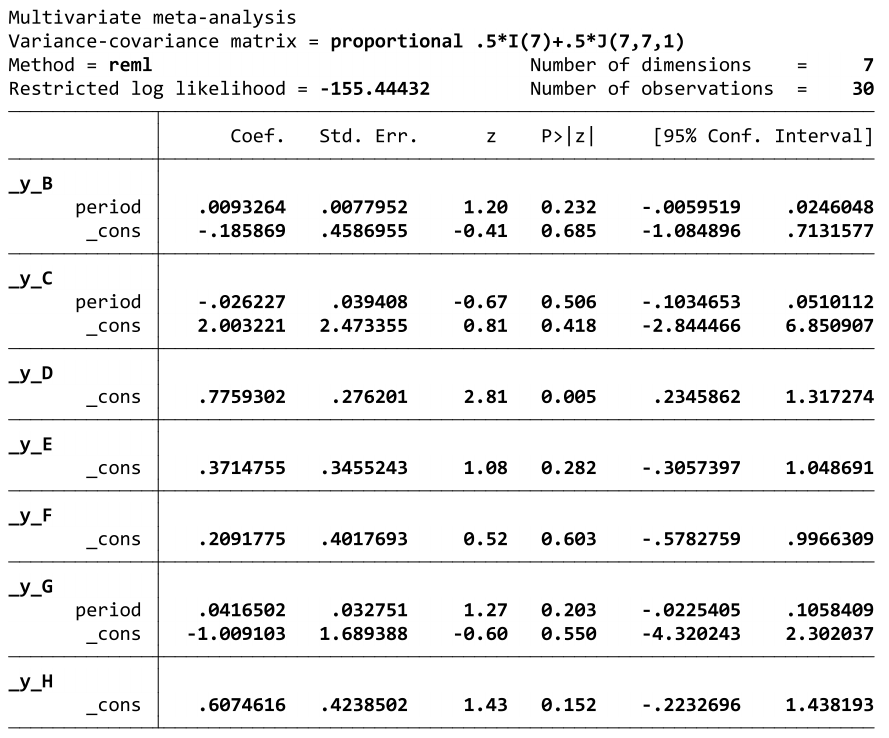


**Table S8.1.3 Meta-regression analysis of covariate as definition period of weaning success in outcome for weaning success**

**B**, Pressure support ventilation (PSV); **C,** Synchronized intermittent mandatory ventilation (SIMV); **D**, Automatic tube compensation (ATC); **E**, Continuous positive airway pressure (CPAP); **F**, Adaptive support ventilation(ASV); **G**: Proportional assist ventilation (PAV); **H**: SmartCare

Proportion of reintubation

*Meta-regression Covariate: Age*

chi2(7) = 4.

Prob > chi2 = 0.4260


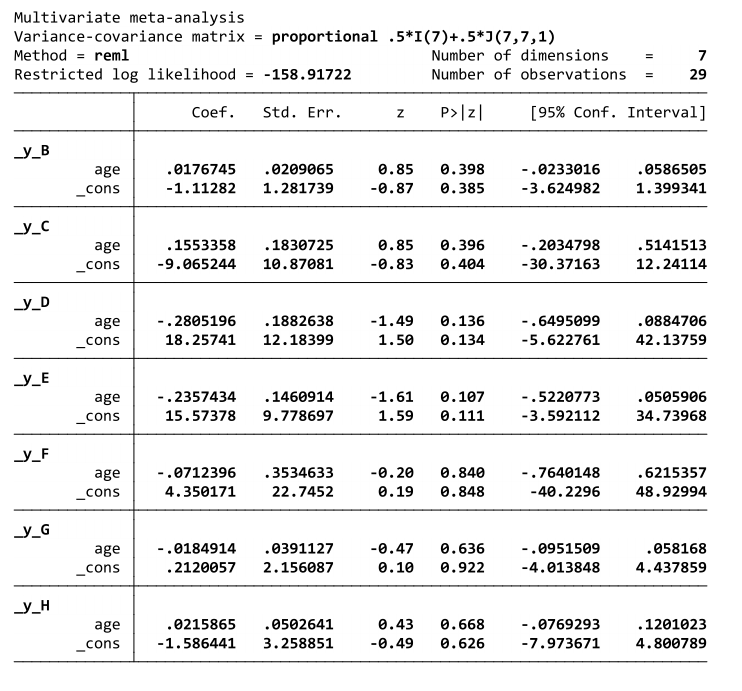


**Table S8.2.1 Meta-regression analysis of covariate as age in outcome for proportion of reintubation**

**B**, Pressure support ventilation (PSV); **C,** Synchronized intermittent mandatory ventilation (SIMV); **D**, Automatic tube compensation (ATC); **E**, Continuous positive airway pressure (CPAP); **F**, Adaptive support ventilation(ASV); **G**: Proportional assist ventilation (PAV); **H**: SmartCare

*Meta-regression Covariate: physiology score (APACHE II): insufficient observations*

*Meta-regression Covariate: baseline duration of mechanical ventilation*

chi2(7) = 5.44

Prob > chi2 = 0.6058


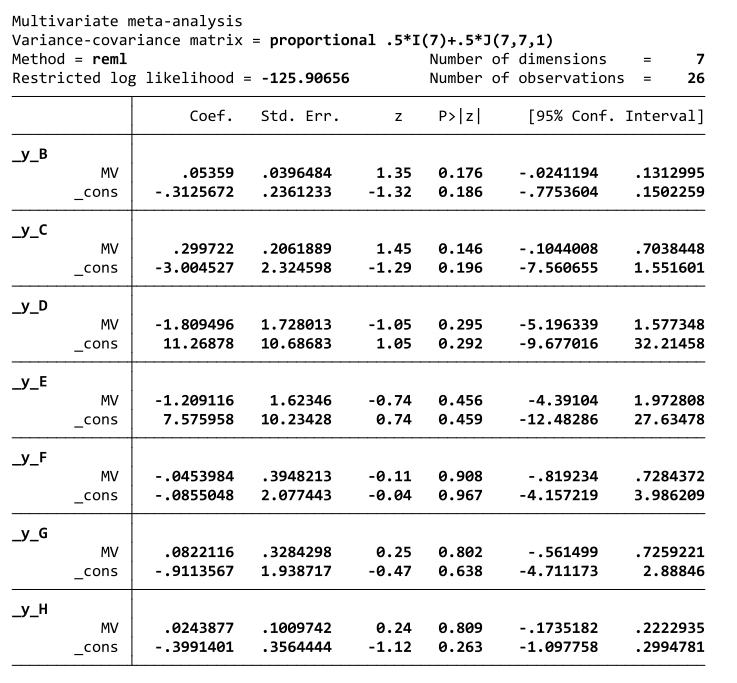


**Table S8.2.2 Meta-regression analysis of covariate as baseline duration of mechanical ventilation in outcome for proportion of reintubation**

**B**, Pressure support ventilation (PSV); **C,** Synchronized intermittent mandatory ventilation (SIMV); **D**, Automatic tube compensation (ATC); **E**, Continuous positive airway pressure (CPAP); **F**, Adaptive support ventilation(ASV); **G**: Proportional assist ventilation (PAV); **H**: SmartCare

*Meta-regression Covariate: Definition period of weaning success*

chi2(2) = 1.66

Prob > chi2 = 0.4357


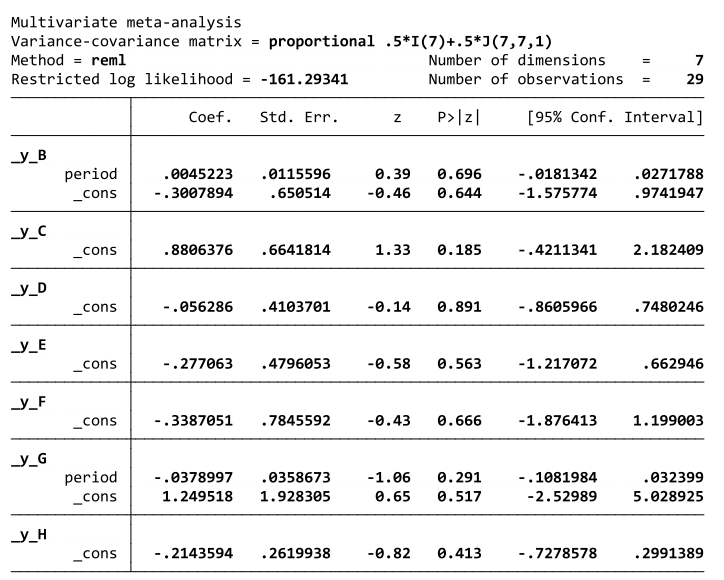


**Table S8.2.3 Meta-regression analysis of covariate as definition period of weaning success in outcome for proportion of reintubation**

**B**, Pressure support ventilation (PSV); **C,** Synchronized intermittent mandatory ventilation (SIMV); **D**, Automatic tube compensation (ATC); **E**, Continuous positive airway pressure (CPAP); **F**, Adaptive support ventilation(ASV); **G**: Proportional assist ventilation (PAV); **H**: SmartCare

Mortality

*Meta-regression Covariate: Age*

chi2(4) = 3.22

Prob > chi2 = 0.5211


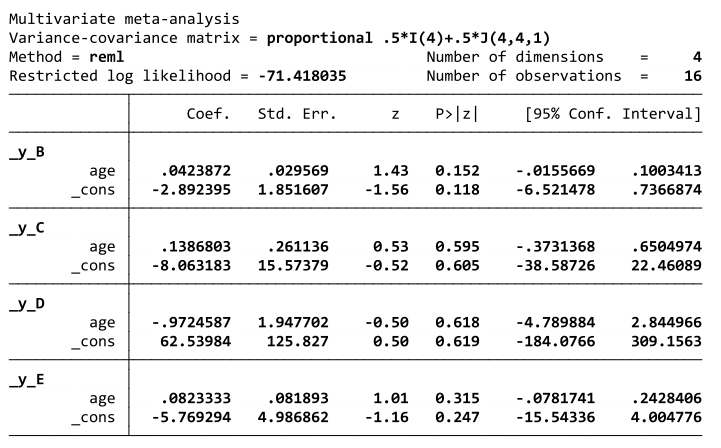


**Table S8.3.1 Meta-regression analysis of covariate as age in outcome for mortality**

**B**, Pressure support ventilation (PSV); **C,** Synchronized intermittent mandatory ventilation (SIMV); **D**, Adaptive support ventilation(ASV); **E**: Proportional assist ventilation (PAV); **F**: SmartCare

*Meta-regression Covariate: physiology score: insufficient observations*

*Meta-regression Covariate: baseline duration of mechanical ventilation*

chi2(4) = 0.62

Prob > chi2 = 0.9603


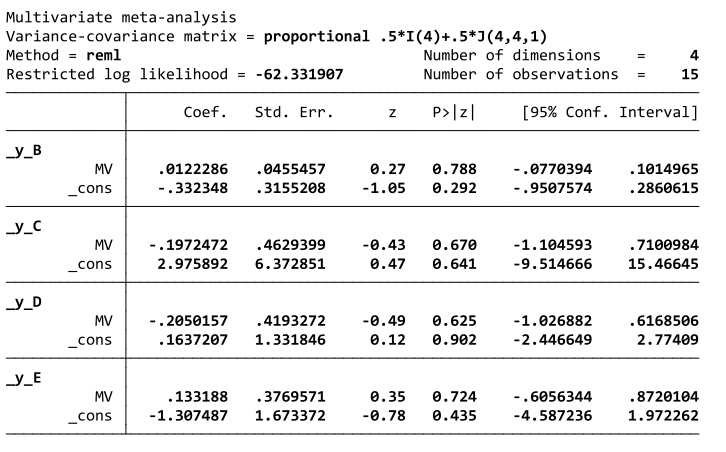


**Table S8.3.2 Meta-regression analysis of covariate as baseline duration of mechanical ventilation in outcome for mortality**

**B**, Pressure support ventilation (PSV); **C,** Synchronized intermittent mandatory ventilation (SIMV); **D**, Adaptive support ventilation(ASV); **E**: Proportional assist ventilation (PAV); **F**: SmartCare

*Meta-regression Covariate: Definition period of weaning success*

chi2(2) = 0.06

Prob > chi2 = 0.9718


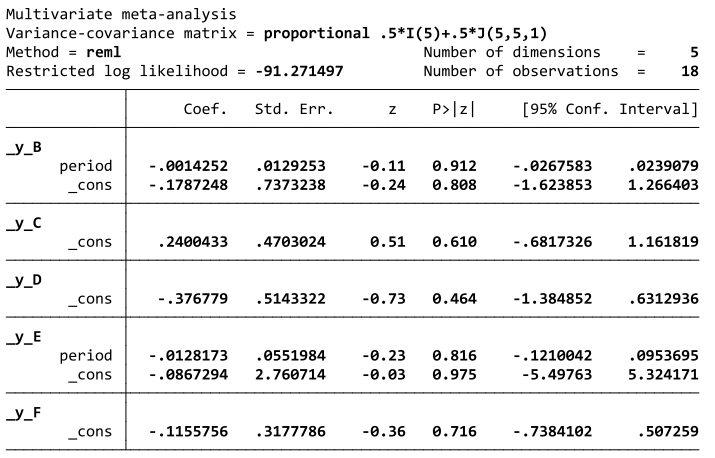


**Table S8.3.3 Meta-regression analysis of covariate as definition period of weaning success in outcome for mortality**

**B**, Pressure support ventilation (PSV); **C,** Synchronized intermittent mandatory ventilation (SIMV); **D**, Automatic tube compensation (ATC); **E**, Continuous positive airway pressure (CPAP); **F**, Adaptive support ventilation(ASV); **G**: Proportional assist ventilation (PAV); **H**: SmartCare

**Table S9. Reference list of included studies**

1. Brochard, L. et al. Comparison of three methods of gradual withdrawal from ventilatory support during weaning from mechanical ventilation. Am J Respir Crit Care Med 150, 896-903, doi:10.1164/ajrccm.150.4.7921460 (1994).
2. Esteban, A. et al. Extubation outcome after spontaneous breathing trials with T-tube or pressure support ventilation. The Spanish Lung Failure Collaborative Group. Am J Respir Crit Care Med 156, 459-465, doi:10.1164/ajrccm.156.2.9610109 (1997).
3. Esteban, A. et al. A Comparison of Four Methods of Weaning Patients from Mechanical Ventilation. New England Journal of Medicine 332, 345-350, doi:10.1056/nejm199502093320601 (1995).
4. Haberthur, C. et al. Extubation after breathing trials with automatic tube compensation, T-tube, or pressure support ventilation. Acta Anaesthesiol Scand 46, 973-979, doi:10.1034/j.1399-6576.2002.460808.x (2002).
5. Koh, Y. et al. Effect of an additional 1-hour T-piece trial on weaning outcome at minimal pressure support. J Crit Care 15, 41-45, doi:10.1053/jcrc.2000.7898 (2000).
6. Koksal, G. M., Sayilgan, C., Sen, O. & Oz, H. The effects of different weaning modes on the endocrine stress response. Critical care (London, England) 8, R31-R34, doi:10.1186/cc2413 (2004).
7. Vitacca, M. et al. Comparison of two methods for weaning patients with chronic obstructive pulmonary disease requiring mechanical ventilation for more than 15 days. Am J Respir Crit Care Med 164, 225-230, doi:10.1164/ajrccm.164.2.2008160 (2001).
8. Matic, I. & Majeric-Kogler, V. Comparison of pressure support and T-tube weaning from mechanical ventilation: randomized prospective study. Croat Med J 45, 162-166 (2004).
9. Y-MMLPY-N, L. A comparison of T-tube and pressure support ventilation for programatic extubation. Biomedical Engineering and Clinical Medicine 5, 277-280 (2005).
10. Cohen, J. et al. Prediction of extubation outcome: a randomised, controlled trial with automatic tube compensation vs. pressure support ventilation. Critical care (London, England) 13, R21-R21, doi:10.1186/cc7724 (2009).
11. Cohen, J. D. et al. Extubation outcome following a spontaneous breathing trial with automatic tube compensation versus continuous positive airway pressure. Crit Care Med 34, 682-686, doi:10.1097/01.Ccm.0000201888.32663.6a (2006).
12. Matic, I. et al. Chronic obstructive pulmonary disease and weaning of difficult-to-wean patients from mechanical ventilation: randomized prospective study. Croat Med J 48, 51-58 (2007).
13. Xirouchaki, N. et al. Proportional assist ventilation with load-adjustable gain factors in critically ill patients: comparison with pressure support. Intensive Care Med 34, 2026-2034, doi:10.1007/s00134-008-1209-2 (2008).
14. Deng, J.-J. & Wang, Q.-L. The comparison of two method to liberation from mechenical ventilation in ICU (ICU zhong liang zhong ji xie tong qi che li mo shi de bi jiao). The Medical Forum(Chinese), doi:10.3969/j.issn.1672-1721.2009.29.010 (2009).
15. Figueroa-Casas, J. B., Montoya, R., Arzabala, A. & Connery, S. M. Comparison between automatic tube compensation and continuous positive airway pressure during spontaneous breathing trials. Respir Care 55, 549-554 (2010).
16. He, H., Chen, Z. & Yang, C. Comparison of two modes of spontaneous breathing trial for weaning from mechanical ventilation. JiangXi Medical Journal (Chinese) 45, 1167-1169 (2010).
17. LI, C., YAN, C.-z. & WANG, Z.-y. Comparison of two weaning methods for patients with chronic obstructive pulmonary disease. Journal of Xinjiang Medical University 2010 May.33(5) (2010).
18. Molina-Saldarriaga, F. J., Fonseca-Ruiz, N. J., Cuesta-Castro, D. P., Esteban, A. & Frutos-Vivar, F. Ensayo de respiración espontánea en pacientes con enfermedad pulmonar obstructiva crónica: presión positiva continua de la vía aérea (CPAP) versus Tubo en T. Medicina Intensiva 34, 453-458, doi:https://doi.org/10.1016/j.medin.2010.03.007 (2010).
19. Bosma, K. J. et al. A Pilot Randomized Trial Comparing Weaning From Mechanical Ventilation on Pressure Support Versus Proportional Assist Ventilation. Crit Care Med 44, 1098-1108, doi:10.1097/ccm.0000000000001600 (2016).
20. Botha, J. et al. Proportional assist ventilation versus pressure support ventilation in weaning ventilation: a pilot randomised controlled trial. Crit Care Resusc 20, 33-40 (2018).
21. Cekmen, N. & Erdemli, O. The comparison of the effects of T-piece and CPAP on hemodynamic parameters, arterial blood gases and success of weaning. Bratisl Lek Listy 112, 512-516 (2011).
22. Chen, F. COPD huan zhe ji xie tong qi che ji fang fa de lin chuang dui zhao yan jiu Medical Innovation of China(Chinese), 28-29, doi:DOI：10.3969/j.issn.1674-4985.2012.28.015 (2012).
23. Chittawatanarat, K., Orrapin, S., Jitkaroon, K., Mueakwan, S. & Sroison, U. An Open Label Randomized Controlled Trial to Compare Low Level Pressure Support and T-piece as Strategies for Discontinuation of Mechanical Ventilation in a General Surgical Intensive Care Unit. Med Arch 72, 51-57, doi:10.5455/medarh.2018.72.51-57 (2018).
24. Elganady, A. A., Beshey, B. N. & Abdelaziz, A. A. H. Proportional assist ventilation versus pressure support ventilation in the weaning of patients with acute exacerbation of chronic obstructive pulmonary disease. Egyptian Journal of Chest Diseases and Tuberculosis 63, 643-650, doi:https://doi.org/10.1016/j.ejcdt.2014.04.001 (2014).
25. Kirakli, C. et al. Adaptive support ventilation for faster weaning in COPD: a randomised controlled trial. Eur Respir J 38, 774-780, doi:10.1183/09031936.00081510 (2011).
26. Mohamed, K. A. E. & El Maraghi, S. k. Role of Adaptive Support Ventilation in Weaning of COPD Patients. Egyptian Journal of Chest Diseases and Tuberculosis 63, 449-454, doi:https://doi.org/10.1016/j.ejcdt.2013.12.017 (2014).
27. Santos Pellegrini, J. A. et al. Pressure-support ventilation or T-piece spontaneous breathing trials for patients with chronic obstructive pulmonary disease - A randomized controlled trial. PLoS One 13, e0202404, doi:10.1371/journal.pone.0202404 (2018).
28. Sasikumar, S., Shanbhag, V., Shenoy, A. & Unnikrishnan, R. Comparison of pressure support and proportional assist ventilation with load-adjustable gain factors for weaning from mechanical ventilation in critically ill patients. European Respiratory Journal 42, P4926 (2013).
29. Subira, C. et al. Effect of Pressure Support vs T-Piece Ventilation Strategies During Spontaneous Breathing Trials on Successful Extubation Among Patients Receiving Mechanical Ventilation: A Randomized Clinical Trial. Jama 321, 2175-2182, doi:10.1001/jama.2019.7234 (2019).
30. Teixeira, S. N. et al. Comparison of Proportional Assist Ventilation Plus, T-Tube Ventilation, and Pressure Support Ventilation as Spontaneous Breathing Trials for Extubation: A Randomized Study. Respir Care 60, 1527-1535, doi:10.4187/respcare.03915 (2015).
31. Zhang, B. & Qin, Y. Z. Comparison of pressure support ventilation and T-piece in determining rapid shallow breathing index in spontaneous breathing trials. Am J Med Sci 348, 300-305, doi:10.1097/maj.0000000000000286 (2014).
32. Burns, K. E., Meade, M. O., Lessard, M. R., Keenan, S. P. & Lellouche, F. Wean Earlier and Automatically with New technology (the WEAN study): a protocol of a multicentre, pilot randomized controlled trial. Trials 10, 81, doi:10.1186/1745-6215-10-81 (2009).
33. Jiang, H., Yu, S. & Wang, L. Comparison of SmartCare and spontaneous breathing trials for weaning old patients with chronic obstructive pulmonary diseases. Chinese Journal of Tuberculosis and Respiratory Disease 8 (2006).
34. Ma, Y., Yang, X., Cao, X. & Ma, X. Comparison of com puter-driven weaning and physician·-directed weaning from m echanical ventilation:arandomizedprospectivestudy. Chinese Journal of Tuberculosis and Respiratory Disease 33, 174-178 (2010).
35. Rose, L., Presneill, J. J., Johnston, L. & Cade, J. F. A randomised, controlled trial of conventional versus automated weaning from mechanical ventilation using SmartCare/PS. Intensive Care Med 34, 1788-1795, doi:10.1007/s00134-008-1179-4 (2008).
36. Schadler, D. et al. Automatic control of pressure support for ventilator weaning in surgical intensive care patients. Am J Respir Crit Care Med 185, 637-644, doi:10.1164/rccm.201106-1127OC (2012).
37. Sulzer, C. F., Chiolero, R., Chassot, P. G., Mueller, X. M. & Revelly, J. P. Adaptive support ventilation for fast tracheal extubation after cardiac surgery: a randomized controlled study. Anesthesiology 95, 1339-1345, doi:10.1097/00000542-200112000-00010 (2001).
38. Taniguchi, C. et al. Smart Care versus respiratory physiotherapy-driven manual weaning for critically ill adult patients: a randomized controlled trial. Crit Care 19, 246, doi:10.1186/s13054-015-0978-6 (2015).
39. Tong, L. & Liu, Y. Research on measures of weaning from mechanical Ventilation-dependency patients with chronic obstructive pulmonary diseases. China Modern Medicine 21 (2014).

**Figure S1. Risk of bias table of included studies**

S1.1 Risk of bias graph


S2.2 Risk of bias summary

**Figure S2. Direct-indirect evidence contribution plot**Weaning success

**Figure S2-1 Direct-indirect evidence contribution plot: Weaning success**

**ASV**, Adaptive support ventilation (ASV); **ATC**, Automatic tube compensation; **CPAP**, Continuous positive airway pressure; **PAV**: Proportional assist ventilation (PAV, including PAV+ mode: PAV with load-adjustable gain factors); **PSV**, Pressure support ventilation; **SIMV,** Synchronized intermittent mandatory ventilation

Proportion requiring reintubation

**Figure S2-2 Direct-indirect evidence contribution plot: Proportion requiring reintubation**

**ASV**, Adaptive support ventilation (ASV); **ATC**, Automatic tube compensation; **CPAP**, Continuous positive airway pressure; **PAV**: Proportional assist ventilation (PAV, including PAV+ mode: PAV with load-adjustable gain factors); **PSV**, Pressure support ventilation; **SIMV,** Synchronized intermittent mandatory ventilation

Mortality

**Figure S2-3 Direct-indirect evidence contribution plot: Mortality**

**ASV**, Adaptive support ventilation (ASV); **ATC**, Automatic tube compensation; **CPAP**, Continuous positive airway pressure; **PAV**: Proportional assist ventilation (PAV, including PAV+ mode: PAV with load-adjustable gain factors); **PSV**, Pressure support ventilation; **SIMV,** Synchronized intermittent mandatory ventilation

**Figure S3. Comparison direct and indirect evidence in network meta-analysis**

Weaning success

**Figure S3-1 Comparison direct and indirect evidence in network meta-analysis: Weaning success**

**ASV**, Adaptive support ventilation (ASV); **ATC**, Automatic tube compensation; **CPAP**, Continuous positive airway pressure; **PAV**: Proportional assist ventilation (PAV, including PAV+ mode: PAV with load-adjustable gain factors); **PSV**, Pressure support ventilation; **SIMV,** Synchronized intermittent mandatory ventilation; **OR**, Odds ratio; **CI**, Confident interval

Proportion requiring reintubation

**Figure S3-2 Comparison direct and indirect evidence in network meta-analysis: Proportion requiring reintubation**

**ASV**, Adaptive support ventilation (ASV); **ATC**, Automatic tube compensation; **CPAP**, Continuous positive airway pressure; **PAV**: Proportional assist ventilation (PAV, including PAV+ mode: PAV with load-adjustable gain factors); **PSV**, Pressure support ventilation; **SIMV,** Synchronized intermittent mandatory ventilation; **OR**, Odds ratio; **CI**, Confident interval

Mortality

**Figure S3-3 Comparison direct and indirect evidence in network meta-analysis: Mortality**

**ASV**, Adaptive support ventilation (ASV); **ATC**, Automatic tube compensation; **CPAP**, Continuous positive airway pressure; **PAV**: Proportional assist ventilation (PAV, including PAV+ mode: PAV with load-adjustable gain factors); **PSV**, Pressure support ventilation; **SIMV,** Synchronized intermittent mandatory ventilation; **OR**, Odds ratio; **CI**, Confident interval

**Figure S4. Subgroup analyses and sensitivity analyses**

**Subgroup analyses**

Subgroup analyses were conducted in accordance with types of endotracheal prosthesis: Endotracheal tube (TOT) and tracheostomy (TQT), patients with underlying COPD, publication year (before and after 2008, the year that first trial conducted with PAV on the purpose of liberation from mechanical ventilation).

However, due to limited trials, subgroup analyses of types of endotracheal prosthesis with TQT could not be performed network meta-analysis.

Figure S4.1 Subgroup of types of endotracheal prosthesis:

*Outcome for weaning success*

**Figure S4.1.1 Subgroup of types of endotracheal prosthesis in outcome for weaning success:**

**ASV**, Adaptive support ventilation; **ATC**, Automatic tube compensation; **CPAP**, Continuous positive airway pressure; **PAV**, Proportional assist ventilation; **PSV**, Pressure support ventilation; **SIMV**, Synchronized intermittent mandatory ventilation; **OR**, Odds ratio; **CI**, Confidence interval; **SUCRA(P-score)**, Surface under the cumulative ranking curve

*Outcome for proportion requiring reintubation*

**Figure S4.1.2 Subgroup of types of endotracheal prosthesis in outcome for proportion requiring reintubation:**

**ASV**, Adaptive support ventilation; **ATC**, Automatic tube compensation; **CPAP**, Continuous positive airway pressure; **PAV**, Proportional assist ventilation; **PSV**, Pressure support ventilation; **SIMV**, Synchronized intermittent mandatory ventilation; **OR**, Odds ratio; **CI**, Confidence interval; **SUCRA(P-score)**, Surface under the cumulative ranking curve

*Outcome for mortality*

**Figure S4.1.3 Subgroup of types of endotracheal prosthesis in outcome for mortality:**

**ASV**, Adaptive support ventilation; **ATC**, Automatic tube compensation; **CPAP**, Continuous positive airway pressure; **PAV**, Proportional assist ventilation; **PSV**, Pressure support ventilation; **SIMV**, Synchronized intermittent mandatory ventilation; **OR**, Odds ratio; **CI**, Confidence interval; **SUCRA(P-score)**, Surface under the cumulative ranking curve

Figure S4.2 Subgroup of patients with underlying COPD:

*Outcome for weaning success*

**Figure S4.2.1 Subgroup of patients with underlying COPD in outcome for weaning success:**

**ASV**, Adaptive support ventilation; **ATC**, Automatic tube compensation; **CPAP**, Continuous positive airway pressure; **PAV**, Proportional assist ventilation; **PSV**, Pressure support ventilation; **SIMV**, Synchronized intermittent mandatory ventilation; **OR**, Odds ratio; **CI**, Confidence interval; **SUCRA(P-score)**, Surface under the cumulative ranking curve

*Outcome for proportion requiring reintubation*

**Figure S4.2.2 Subgroup of patients with underlying COPD in outcome for proportion requiring reintubation:**

**ASV**, Adaptive support ventilation; **ATC**, Automatic tube compensation; **CPAP**, Continuous positive airway pressure; **PAV**, Proportional assist ventilation; **PSV**, Pressure support ventilation; **SIMV**, Synchronized intermittent mandatory ventilation; **OR**, Odds ratio; **CI**, Confidence interval; **SUCRA(P-score)**, Surface under the cumulative ranking curve

*Outcome for mortality*

**Figure S4.2.3 Subgroup of patients with underlying COPD in outcome for mortality:**

**ASV**, Adaptive support ventilation; **ATC**, Automatic tube compensation; **CPAP**, Continuous positive airway pressure; **PAV**, Proportional assist ventilation; **PSV**, Pressure support ventilation; **SIMV**, Synchronized intermittent mandatory ventilation; **OR**, Odds ratio; **CI**, Confidence interval; **SUCRA(P-score)**, Surface under the cumulative ranking curve

Figure S4.3 Subgroup of publication year:

*Outcome for weaning success*

**Figure S4.3.1 Subgroup of patients with publication year before 2008 in outcome for weaning success:**

**ASV**, Adaptive support ventilation; **ATC**, Automatic tube compensation; **CPAP**, Continuous positive airway pressure; **PAV**, Proportional assist ventilation; **PSV**, Pressure support ventilation; **SIMV**, Synchronized intermittent mandatory ventilation; **OR**, Odds ratio; **CI**, Confidence interval; **SUCRA(P-score)**, Surface under the cumulative ranking curve

**Figure S4.3.2 Subgroup of patients with publication year after 2008 in outcome for weaning success:**

**ASV**, Adaptive support ventilation; **ATC**, Automatic tube compensation; **CPAP**, Continuous positive airway pressure; **PAV**, Proportional assist ventilation; **PSV**, Pressure support ventilation; **SIMV**, Synchronized intermittent mandatory ventilation; **OR**, Odds ratio; **CI**, Confidence interval; **SUCRA(P-score)**, Surface under the cumulative ranking curve

*Outcome for proportion requiring reintubation*


**Figure S4.3.3 Subgroup of patients with publication year before 2008 in outcome for proportion requiring reintubation:**

**ASV**, Adaptive support ventilation; **ATC**, Automatic tube compensation; **CPAP**, Continuous positive airway pressure; **PAV**, Proportional assist ventilation; **PSV**, Pressure support ventilation; **SIMV**, Synchronized intermittent mandatory ventilation; **OR**, Odds ratio; **CI**, Confidence interval; **SUCRA(P-score)**, Surface under the cumulative ranking curve

**Figure S4.3.4 Subgroup of patients with publication year after 2008 in outcome for proportion requiring reintubation:**

**ASV**, Adaptive support ventilation; **ATC**, Automatic tube compensation; **CPAP**, Continuous positive airway pressure; **PAV**, Proportional assist ventilation; **PSV**, Pressure support ventilation; **SIMV**, Synchronized intermittent mandatory ventilation; **OR**, Odds ratio; **CI**, Confidence interval; **SUCRA(P-score)**, Surface under the cumulative ranking curve

*Mortality*

**Figure S4.3.5 Subgroup of patients with publication year before 2008 in outcome for mortality:**

**ASV**, Adaptive support ventilation; **ATC**, Automatic tube compensation; **CPAP**, Continuous positive airway pressure; **PAV**, Proportional assist ventilation; **PSV**, Pressure support ventilation; **SIMV**, Synchronized intermittent mandatory ventilation; **OR**, Odds ratio; **CI**, Confidence interval; **SUCRA(P-score)**, Surface under the cumulative ranking curve

**Figure S4.3.6 Subgroup of patients with publication year after 2008 in outcome for mortality:**

**ASV**, Adaptive support ventilation; **ATC**, Automatic tube compensation; **CPAP**, Continuous positive airway pressure; **PAV**, Proportional assist ventilation; **PSV**, Pressure support ventilation; **SIMV**, Synchronized intermittent mandatory ventilation; **OR**, Odds ratio; **CI**, Confidence interval; **SUCRA(P-score)**, Surface under the cumulative ranking curve

**Sensitivity Analyses**

Sensitivity analyses were based on the represented the greatest risk-of-bias to the validity of study ﬁndings including omission of small trials (<25th percentiles), exclusion of trials with high risk-of-bias.

Figure S4.5 Sensitivity Analyses of omission of small trials

*Weaning success*

**Figure S4.5.1 Sensitivity analyses of omission of small trials in outcome for weaning success:**

**ASV**, Adaptive support ventilation; **ATC**, Automatic tube compensation; **CPAP**, Continuous positive airway pressure; **PAV**, Proportional assist ventilation; **PSV**, Pressure support ventilation; **SIMV**, Synchronized intermittent mandatory ventilation; **OR**, Odds ratio; **CI**, Confidence interval; **SUCRA(P-score)**, Surface under the cumulative ranking curve

*Proportion requiring reintubation*

**Figure S4.5.2 Sensitivity analyses of omission of small trials in outcome for proportion requiring reintubation:**

**ASV**, Adaptive support ventilation; **ATC**, Automatic tube compensation; **CPAP**, Continuous positive airway pressure; **PAV**, Proportional assist ventilation; **PSV**, Pressure support ventilation; **SIMV**, Synchronized intermittent mandatory ventilation; **OR**, Odds ratio; **CI**, Confidence interval; **SUCRA(P-score)**, Surface under the cumulative ranking curve

*Mortality*

**Figure S4.5.3 Sensitivity analyses of omission of small trials in outcome for mortality:**

**ASV**, Adaptive support ventilation; **ATC**, Automatic tube compensation; **CPAP**, Continuous positive airway pressure; **PAV**, Proportional assist ventilation; **PSV**, Pressure support ventilation; **SIMV**, Synchronized intermittent mandatory ventilation; **OR**, Odds ratio; **CI**, Confidence interval; **SUCRA(P-score)**, Surface under the cumulative ranking curve

Figure S4.6 Sensitivity Analyses of exclusion trials with high risk-of-bias

*Weaning success*

**Figure S4.6.1 Sensitivity analyses of exclusion trials with high risk-of-bias in outcome for weaning success:**

**ASV**, Adaptive support ventilation; **ATC**, Automatic tube compensation; **CPAP**, Continuous positive airway pressure; **PAV**, Proportional assist ventilation; **PSV**, Pressure support ventilation; **SIMV**, Synchronized intermittent mandatory ventilation; **OR**, Odds ratio; **CI**, Confidence interval; **SUCRA(P-score)**, Surface under the cumulative ranking curve

*Proportion requiring reintubation*

**Figure S4.6.2 Sensitivity analyses of exclusion trials with high risk-of-bias in outcome for proportion requiring reintubation:**

**ASV**, Adaptive support ventilation; **ATC**, Automatic tube compensation; **CPAP**, Continuous positive airway pressure; **PAV**, Proportional assist ventilation; **PSV**, Pressure support ventilation; **SIMV**, Synchronized intermittent mandatory ventilation; **OR**, Odds ratio; **CI**, Confidence interval; **SUCRA(P-score)**, Surface under the cumulative ranking curve

*Mortality*

**Figure S4.6.3 Sensitivity analyses of exclusion trials with high risk-of-bias in outcome for mortality:**

**ASV**, Adaptive support ventilation; **ATC**, Automatic tube compensation; **CPAP**, Continuous positive airway pressure; **PAV**, Proportional assist ventilation; **PSV**, Pressure support ventilation; **SIMV**, Synchronized intermittent mandatory ventilation; **OR**, Odds ratio; **CI**, Confidence interval; **SUCRA(P-score)**, Surface under the cumulative ranking curve

**Figure S5. Comparison-adjusted funnel plots and Egger’s test**

Weaning success

**Figure S5.1 Comparison-adjusted funnel plot in outcome for weaning success**

Proportion of reintubation

**Figure S5.2 Comparison-adjusted funnel plot in outcome for proportion of reintubation**

Mortality

**Figure S5.3 Comparison-adjusted funnel plot in outcome for mortality**
